# Association between the *p53* polymorphisms and cervical cancer risk: an updated meta-analysis

**DOI:** 10.3389/fonc.2025.1461737

**Published:** 2025-02-21

**Authors:** Xi-Qin Zhang, Xiao-Hui Bai, Hui-Zhen Zhang, Xiao-Feng He

**Affiliations:** ^1^ Department of Gynaecology, Heping Hospital Affiliated to Changzhi Medical College, Changzhi, China; ^2^ Guilin Medical University, Guilin, Guangxi, China; ^3^ Institute of Evidence-Based Medicine, Heping Hospital Affiliated to Changzhi Medical College, Changzhi, China

**Keywords:** p53, polymorphism, risk, cervical cancer, meta-analysis

## Abstract

**Background:**

The association of the *p53* rs1042522 and rs17878362 polymorphisms with cervical cancer risk has been reported in several published original studies and meta-analyses. However, the conclusions of these studies were contradictory. Consequently, we conducted an updated meta-analysis to further validate these debates.

**Objective:**

To evaluate the association between the *p53* rs1042522 and rs17878362 polymorphisms and cervical cancer risk.

**Materials and Methods:**

PubMed, Medline, Ovid, Embase, CNKI, and China Wanfang databases were searched. Association was assessed using odds ratio (OR) with 95% confidence interval (CI). Moreover, the false-positive reporting probability (FPRP), Bayesian false-finding probability (BFDP), and Venice criteria were used to assess the credibility of statistically significant association.

**Results:**

A significantly decreased cervical cancer risk was revealed for the p53 rs1042522 polymorphism (Pro/Pro +Arg/Pro vs. Arg/Arg: OR = 0.79, 95% CI = 0.71-0.87; Pro/Pro vs. Arg/Arg: OR = 0.80, 95% CI = 0.70-0.91; Arg/Pro vs. Arg/Arg: OR = 0.78, 95% CI = 0.71-0.86; Pro vs. Arg: OR = 0.87, 95% CI = 0.81-0.93) in overall analysis and several subgroup analyses, such as in Caucasians, Asians, Indians, and so on. However, no significant association was found between the p53 rs17878362 polymorphism and cervical cancer risk. Despite these statistically significant results, reliability analysis using FPRP, BFDP, and Venice criteria deemed all associations “unreliable”.

**Conclusions:**

After considering the reliability of the results, this study indicates that the p53 rs1042522 polymorphism is not associated with the cervical cancer risk.

## Introduction

According to global cancer statistics, cervical cancer is classified by World Health Organization (WHO) as the second most prevalent malignant tumor of the female reproductive system, following breast cancer (1). In many developing countries, there continues to be a rise in the prevalence of cervical cancer. The latest statistics reveal that approximately 3.11 million new cases of cervical cancer occur worldwide each year, with around 570,000 cases being diagnosed annually (2, 3). Furthermore, there is an increasing trend in the occurrence of cervical cancer among young women. The *p53* gene plays a crucial role as a tumor suppressor gene and possesses various biological functions such as inhibiting tumor cell growth and inducing cell cycle arrest at G1 phase. It also promotes programmed cell death after DNA damage and safeguards genetic stability.

The p53 gene, situated on the short arm of chromosome 17, holds a pivotal position as a tumor suppressor gene. Its structure encompasses multiple functional domains, including those for transcription activation and DNA binding. The p53 exerts its regulatory influence on the expression of specific genes in response to a variety of stimuli, operating through both transcriptional and non-transcriptional mechanisms. Mutations in p53 have the potential to disrupt its vital functions, encompassing cell cycle regulation, DNA repair, and the induction of apoptosis, thereby facilitating the onset and progression of tumorigenesis (4). The most common locus for variation is the *p53* codon 72 (rs1042522). This mutation leads to functional inactivation of coding proteins *p53* Arg and *p53* Pro and may contribute to tumorigenesis through various mechanisms. Recent investigations on cervical cancer have revealed that mutations in host *p53* gene polymorphisms play a significant role in its onset and progression. Furthermore, research suggests that individuals carrying the Arg form of *p53* are more susceptible to cervical cancer compared to those carrying Pro (5, 6, 15). Therefore, understanding these genetic variations can provide valuable insights into the development and management strategies for this disease.

Many studies reported the association between the *p53* codon 72 (rs1042522) and IVS3 16 bp (rs17878362) and cervical cancer risk. However, this association remained a subject of controversy. One hundred and twenty-three articles (7–129) evaluated the relationship between the *p53* codon 72 (rs1042522) and IVS3 16 bp (rs17878362) and cervical cancer risk, yet these findings were inconsistent. Furthermore, previously published meta-analyses did not use the false positive reporting probability (FPRP) (137), Bayesian error detection probability (BFDP) (138), and Venice criteria (139) to assess the credibility of the pooled results (7–15). Therefore, we conducted an updated meta-analysis to further evaluate the above issues.

## Materials and methods

This meta-analysis was conducted according to the Preferred Reporting Items for Systematic Reviews and Meta-Analyses (PRISMA) statement (130).

### Search strategy

PubMed, Medline, Embase, China National Knowledge Network (CNKI), and China Wanfang Databases were used for literature retrieval. The search strategies are as follows (“p53” OR “ tp53 “or” tp-53 “or” p-53 “) and (“ polymorphism “or” variability “or” mutation “or” gene “or” NP “) and (“ cervical “or” cervix “). Literature searches were conducted until October 31, 2023. In addition, a careful review of the reference list of published meta-analyses was conducted to spot all eligible studies.

### Selection criteria

Inclusion criteria were as follows: (1) case-control or cohort studies, (2) associations were evaluated between *p53* rs1042522 and rs17878362 polymorphisms and risk of cervical cancer; (3) detailed genotype data or odds ratios (OR) and their corresponding 95% confidence intervals (CI). Exclusion criteria are as follows: (1) animal experiments or overlapping studies; (2) case reports, abstracts, reviews, letters, and meta-analyses; (3) insufficient genotype data or unavailable for studies.

### Data extraction and quality assessment

Two researchers screened all the literatures according to the inclusion and exclusion criteria. Once variations exist and no accord are often reached once discussion, the other author collected the data once more, and at last the three authors can check and ensure along. The following data was extracted: year of publication, first author, country, region, source of case *p53* genotyping materials, recruitment source, genotype management cluster, total sample size, matching, genotype distribution, etc.

After comprehensively considering the characteristics of the articles, the quality evaluation of all the included literatures was conducted according to some criteria (such as HWE, control matching, certainty, sample size, etc.), as shown in **Supplementary Table S1**. In the control group, we applied the goodness-fit Chi-square test to analyze the Hardy-Weinberg balance (HWE) for eligible studies with complete genotype data. *P* ≥ 0.05 was defined as HWE, and *P* < 0.05 was considered as Hardy–Weinberg disequilibrium (HWD) (131). The highest score was 23, and the eligible studies that met both scoring ≥16 and HWE compliant were considered as high-quality (**Supplementary Table S6**). If there is a disagreement on the score, it is assessed again by a superior author.

### Statistical analysis

Association was evaluated applying the following five genetic models: (1) dominant model (rs1042522: Pro/Pro + Arg/Pro vs Arg/Arg, rs17878362: A2/A2+ A1/A2 vs. A1/A1); (2) recessive model (rs1042522: Pro/Pro vs Arg/Arg + Arg/Pro, rs17878362: A2/A2 vs. A1/A1+ A1/A2); (3) homozygous model (rs1042522: Pro/Pro vs Arg/Arg, rs17878362: A2/A2 vs. A1/A1; (4) codominance model (rs1042522: Arg/Pro vs Arg/Arg, rs17878362: A1/A2 vs. A1/A1); (5) allele model (rs1042522: Pro vs Arg, rs17878362: A1 vs. A2). If the *P* < 0.05 and/or *I^2^* > 50%, indicating significant heterogeneity, a random-effects model was used (132). Instead, a fixed-effects model was used. The sources of heterogeneity were assessed using meta-regression analysis (133). Subgroups were created based on race, region, matching situation, and source of controls. Sensitivity analyses were conducted by individually excluding each study or by excluding studies with both low quality and HWD. Egger’s test (134) and Begg’s test (135) were performed to evaluate potential publication bias. In case of publication bias, a non-parametric “trim and fill” approach (136) was employed to estimate and supplement the number of missing studies. All statistical analyses for this meta-analysis were calculated using STATA code version 12.0 (STATA Corp, College Station, TX, USA).

FPRP, BFDP, and Venetian criteria (139) were utilized to assess the confidence levels for statistically significant associations. Associations meeting the following criteria were considered as highly credible: 1) statistically significant associations observed in at least two genetic models; 2) *I^2^* < 50%; 3) FPRP < 0.2 and BFDP < 0.8; 4) statistical power >80%.

## Result

**Figure 1:**
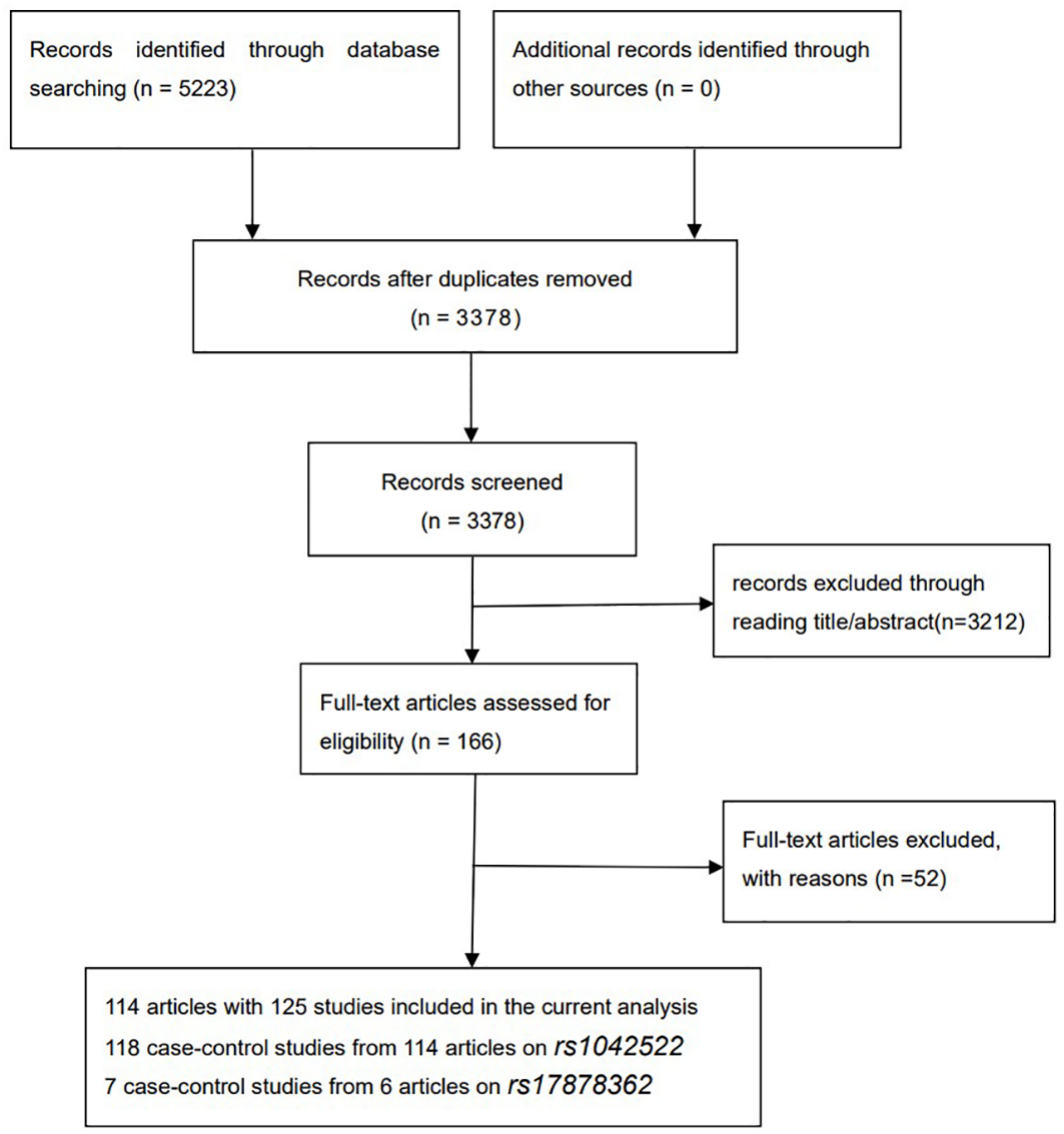
Flow diagram of the literature search.

According to the pre-search methodology employed in this study (**Figure 1**), a total of 5,223 relevant articles were initially identified. After eliminating duplicates from these records, a final set of 3,378 unique publications remained. Subsequently, during the title and abstract screening process, a further 3,212 papers were excluded. Following a thorough full-text review, 22 additional articles were removed due to duplicate or unavailable data, and 30 papers were discarded because of poor quality control. Thus, the final analysis included 114 studies (**supplementary Table S4**-**S5**, **Figure 1**) comprising 125 independent investigations, encompassing a total combined sample size of 13,319 cases and 19,959 controls. As shown in **Supplementary Tables S4**-**S5**, *p53* rs1042522 was reported in 118 studies (12,655 cases and 19,272 controls), while *p53* rs17878362 was reported in seven studies (664 cases and 687 controls). Furthermore, among these studies, there were 37 articles of low quality and 77 articles of high quality for *p53* rs1042522; whereas for *p53* rs17878362, one article was classified as low quality and five articles as high quality (**Supplementary Table S6**). The complete characteristics and genotype frequencies of the literature included are presented in **Supplementary Table S4**-**S5**.

### Quantitative synthesis

#### *P53 rs1042522* polymorphism and cervical cancer

**Figure 2:**
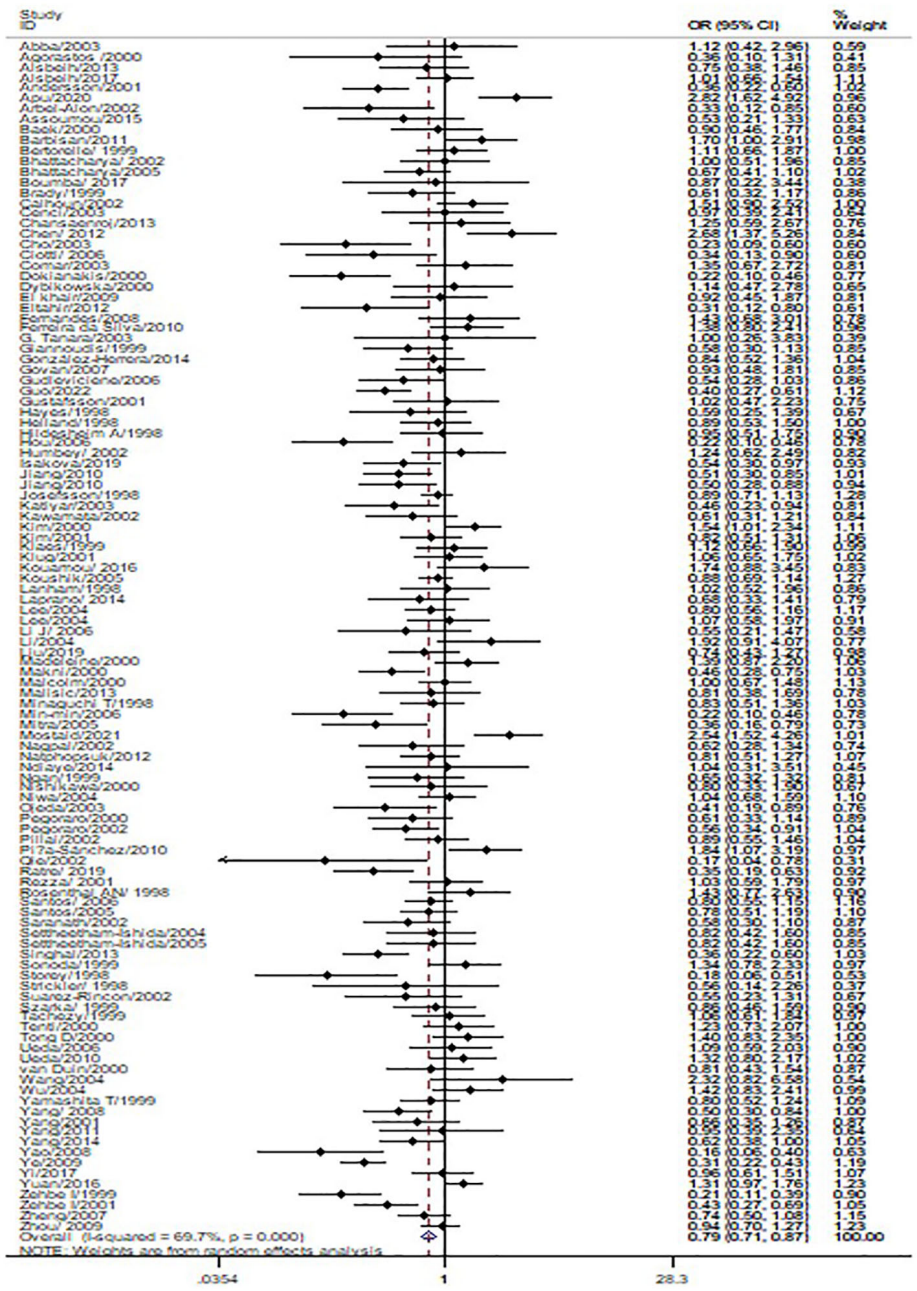
Forest map of the correlation between *p53 rs1042522* polymorphism and cervical cancer in overall analysis (Pro Pro + Arg Pro vs. Arg Arg).

**Figure 3:**
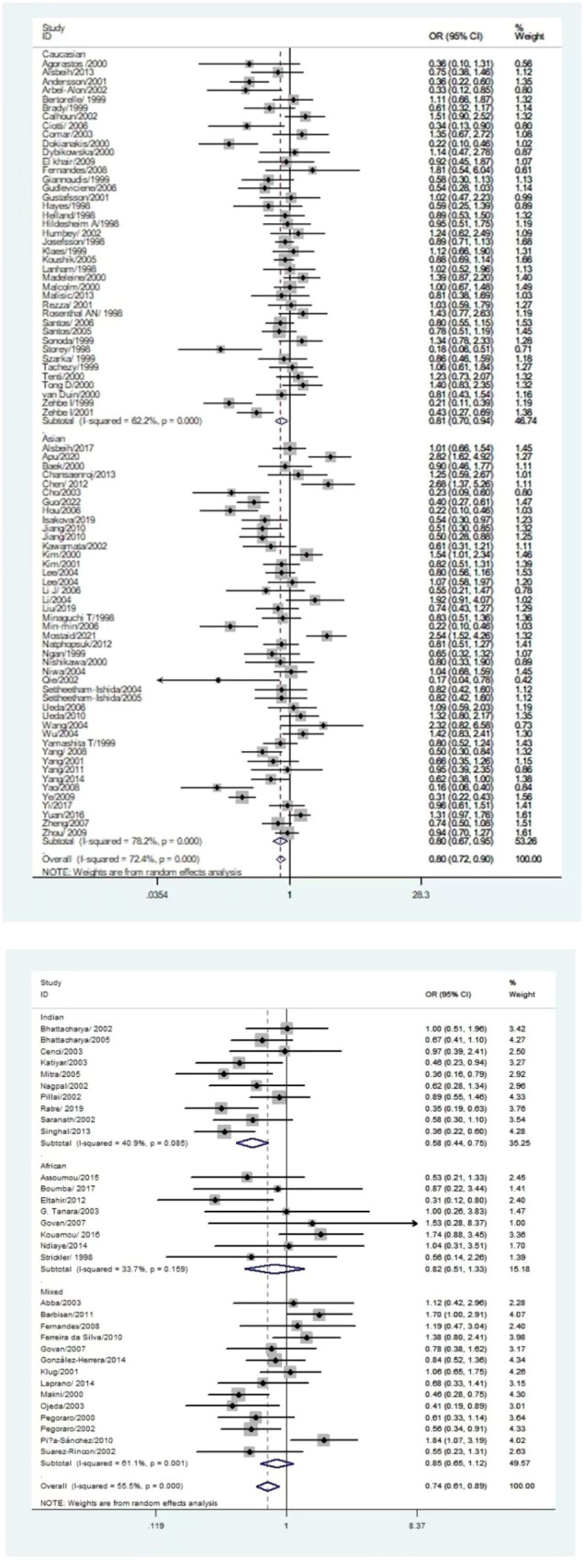
Forest map of the correlation of between *p53 rs1042522* polymorphism and cervical cancer in the ethnicity group analysis forest map (Pro Pro + Arg Pro vs. Arg Arg).

The *p53 rs1042522* polymorphism was significantly associated with a reduced risk of cervical cancer (Pro/Pro +Arg/Pro vs. Arg/Arg: OR = 0.79, 95% CI = 0.71-0.87; Pro/Pro vs. Arg/Arg: OR = 0.80, 95% CI = 0.70-0.91; Arg/Pro vs. Arg/Arg: OR = 0.78, 95% CI = 0.71-0.86; Pro vs. Arg: OR = 0.87, 95% CI = 0.81-0.93, **Table 1**, **Figure 2**) in overall analysis. Moreover, a significantly reduced cervical cancer risk was also observed in Caucasians (Pro/Pro +Arg/Pro vs. Arg/Arg: OR = 0.81, 95% CI = 0.70-0.94; Pro/Pro vs. Arg/Arg: OR = 0.84, 95% CI = 0.73-0.98; Arg/Pro vs. Arg/Arg: OR = 0.81, 95% CI = 0.70-0.94; Pro vs. Arg: OR = 0.86, 95% CI = 0.77-0.96, **Table 1**, **Figure 3**), Asians (Pro/Pro +Arg/Pro vs. Arg/Arg: OR = 0.80, 95% CI = 0.67-0.95; Arg/Pro vs. Arg/Arg: OR = 0.78, 95% CI = 0.66-0.93; Pro vs. Arg: OR = 0.89, 95% CI = 0.79-0.99, **Table 1**, **Figure 3**), Indians (Pro/Pro +Arg/Pro vs. Arg/Arg: OR = 0.57, 95% CI = 0.47-0.70; Arg/Pro vs. Arg/Arg: OR = 0.60, 95% CI = 0.48-0.73, **Table 1**, **Figure 3**), and mixed population (Pro/Pro vs. Arg/Pro + Arg/Arg: OR = 0.81, 95% CI = 0.68-0.98; Pro/Pro vs. Arg/Arg: OR = 0.73, 95% CI = 0.57-0.92; Pro vs. Arg: OR = 0.88, 95% CI = 0.79-0.98, **Table 1**, **Figure 3**). However, no significant association was found between *p53* rs1042522 polymorphism and cervical cancer risk in Africans. Furthermore, significantly reduced risk of cervical cancer was observed in Europe (Pro/Pro +Arg/Pro vs. Arg/Arg: OR = 0.77, 95% CI = 0.65-0.92; Pro/Pro vs. Arg/Arg: OR = 0.84, 95% CI = 0.7-0.99; Arg/Pro vs. Arg/Arg: OR = 0.76, 95% CI = 0.64-0.91; Pro vs. Arg: OR = 0.84, 95% CI = 0.74-0.96, **Table 1**), East Asians (Pro/Pro +Arg/Pro vs. Arg/Arg: OR = 0.74, 95% CI = 0.61-0.90; Pro/Pro vs. Arg/Arg: OR = 0.76, 95% CI = 0.62-0.94; Arg/Pro vs. Arg/Arg: OR = 0.72, 95% CI = 0.59-0.88; Pro vs. Arg: OR = 0.84, 95% CI = 0.75-0.95, **Table 1**), and Africa (Pro/Pro +Arg/Pro vs. Arg/Arg: OR = 0.75, 95% CI = 0.59-0.95; Pro/Pro vs. Arg/Arg: OR = 0.69, 95% CI = 0.48-0.98, **Table 1**). Then, we observed that the *p53 rs1042522* polymorphism reduced the risk of cervical cancer in the matching studies (Pro/Pro +Arg/Pro vs. Arg/Arg: OR = 0.78, 95% CI = 0.68-0.90; Pro/Pro vs. Arg/Arg: OR = 0.75, 95% CI = 0.63-0.90; Arg/Pro vs. Arg/Arg: OR = 0.79, 95% CI = 0.68-0.91; Pro vs. Arg: OR = 0.88, 95% CI = 0.80-0.97, **Table 1**) and non-matching studies (Pro/Pro +Arg/Pro vs. Arg/Arg: OR = 0.79, 95% CI = 0.68-0.91; Pro/Pro vs. Arg/Arg: OR = 0.83, 95% CI = 0.74-0.94; Arg/Pro vs. Arg/Arg: OR = 0.78, 95% CI = 0.68-0.90; Pro vs. Arg: OR = 0.86, 95% CI =0.77-0.96, **Table 1**). Finally, we obtained a significant association in health control population (Pro/Pro +Arg/Pro vs. Arg/Arg: OR = 0.80, 95% CI = 0.69-0.92; Pro/Pro vs. Arg/Arg: OR = 0.80, 95% CI = 0.67-0.95; Arg/Pro vs. Arg/Arg: OR = 0.81, 95% CI = 0.70-0.93; Pro vs. Arg: OR = 0.88, 95% CI =0.8-0.98, **Table 1**) and non-cancer control population (Pro/Pro +Arg/Pro vs. Arg/Arg: OR = 0.77, 95% CI = 0.68-0.88; Pro/Pro vs. Arg/Arg: OR = 0.8, 95% CI = 0.66-0.97; Arg/Pro vs. Arg/Arg: OR = 0.76, 95% CI = 0.66-0.87; Pro vs. Arg: OR = 0.86, 95% CI =0.76-0.95, **Table 1**). The results of sensitivity analysis showed no significant changes in this study. Furthermore, Egger’s test and Begg’s funnel plot confirmed the absence of publication bias (Pro/Pro + Arg/Pro vs. Arg/Arg: *P* = 0.06; Pro/Pro vs. Arg/Pro + Arg/Arg: *P* = 0.386; Pro/Pro vs. Arg/Arg: *P* = 0.673; Arg/Pro vs. Arg/Arg: p=0.091; Pro vs. Arg: *P* = 0.91). In the overall analysis, the results for the Pro Pro +Arg Pro vs. Arg Arg models did not change (data not shown), suggesting that more studies could not change the pooled results (**Figure 5**).

**Table 1:** Meta-analysis of the association of *p53 rs1042522* polymorphism with risk of cervical cancer.

Variable	n (Cases/Controls)	Pro/Pro +Arg/Pro *vs.* Arg/Arg		Pro/Pro *vs.* Arg/Pro + Arg/Arg		Pro/Pro *vs.* Arg/Arg		Arg/Pro *vs*. Arg/Arg		Pro *vs.* Arg	
		OR (95% CI)	*P_h_/I^2^* (%)	OR (95% CI)	*P_h_/I^2^* (%)	OR (95% CI)	*P_h_/I^2^* (%)	OR (95% CI)	*P_h_/I^2^* (%)	OR (95% CI)	*P_h_/I^2^* (%)
Overall	114 (12655/19272)	**0.79(0.71-0.87)**	<0.001/69.7	0.92(0.82-1.03)	<0.001/56.6	**0.80(0.70-0.91)**	<0.001/58.4	**0.78(0.71-0.86)**	<0.001/65.2	**0.87(0.81-0.93)**	<0.001/71.5
Ethnicity											
Caucasian	40 (4020/7676)	**0.81(0.70-0.94)**	<0.001/62.2	0.88(0.76-1.01)	0.039/30.3	**0.84(0.73-0.98)**	0.063/26.9	**0.81(0.70-0.94)**	<0.001/57.4	**0.86(0.77-0.96)**	<0.001/61.5
Asian	44 (5663/7610)	**0.80(0.67-0.95)**	<0.001/78.2	0.94(0.80-1.11)	<0.001/58.9	0.83(0.67-1.02)	<0.001/68.5	**0.78(0.66-0.93)**	<0.001/75	**0.89(0.79-0.99)**	<0.001/77.3
Indian	10 (1227/1924)	**0.57(0.47-0.70)**	0.085/41	0.92(0.53-1.59)	<0.001/86.7	0.64(0.35-1.16)	<0.001/81.9	**0.60(0.48-0.73)**	0.756/0	0.78(0.57-1.06)	<0.001/85.1
African	8 (367/378)	0.84(0.59-1.21)	0.159/33.7	1.08(0.77-1.51)	0.068/46.8	0.78(0.50-1.22)	0.133/37.1	0.82(0.55-1.23)	0.389/5.4	0.98(0.67-1.44)	0.009/62.7
Mixed	14 (1378/2314)	0.85(0.65-1.12)	0.001/61.1	**0.81(0.68-0.98)**	0.248/18.9	**0.73(0.57-0.92)**	0.480/0	0.88(0.65-1.20)	<0.001/66.2	**0.88(0.79-0.98)**	0.090/35.7
Region											
Europe	32 (3118/6007)	**0.77(0.65-0.92)**	<0.001/65.3	0.93(0.79-1.10)	0.253/13.4	**0.84(0.70-0.99)**	0.09/26.2	**0.76(0.64-0.91)**	<0.001/60	**0.84(0.74-0.96)**	<0.001/62.9
South Asia	18 (2219/2360)	0.83(0.63-1.08)	<0.001/72.3	1.04(0.74-1.45)	<0.001/79.9	0.88(0.59-1.31)	<0.001/78.6	0.80(0.64-1.00)	0.003/54.7	0.96(0.77-1.19)	<0.001/83.3
East Asia	36 (4671/6544)	**0.74(0.61-0.90)**	<0.001/77.2	0.90(0.76-1.06)	<0.001/52.3	**0.76(0.62-0.94)**	<0.001/63.1	**0.72(0.59-0.88)**	<0.001/76.5	**0.84(0.75-0.95)**	<0.001/72.4
Africa	10 (933/1160)	**0.75(0.59-0.95)**	0.174/29.4	0.88(0.66-1.18)	0.052/46.5	**0.69(0.48-0.98)**	0.165/30.5	0.78(0.60-1.01)	0.458/0	0.88(0.71-1.10)	0.009/58.8
South America	12 (974/1941)	0.95(0.71-1.27)	0.002/61.9	0.91(0.69-1.19)	0.139/31.5	0.90(0.67-1.20)	0.603/0	0.96(0.68-1.36)	<0.001/70.4	0.96(0.85-1.09)	0.153/29.9
North America	5 (717/1098)	0.87(0.53-1.40)	0.003/75.1	0.78(0.31-1.98)	<0.001/85.9	0.76(0.28-2.05)	<0.001/85.2	0.99(0.80-1.22)	0.471/0	0.82(0.47-1.42)	<0.001/90.1
Matching											
YES	58 (7490/10883)	**0.78(0.68-0.90)**	<0.001/73.9	0.90(0.77-1.05)	<0.001/65.5	**0.75(0.63-0.90)**	<0.001/64.2	**0.79(0.68-0.91)**	<0.001/70.9	**0.88(0.80-0.97)**	<0.001/74.3
NR	56 (5165/8389)	**0.79(0.68-0.91)**	<0.001/64.3	0.93(0.84-1.04)	0.001/41.7	**0.83(0.74-0.94)**	<0.001/49.8	**0.78(0.68-0.90)**	<0.001/57.4	**0.86(0.77-0.96)**	<0.001/68.3
Source of controls											
Healthy	55 (6946/10745)	**0.80(0.69-0.92)**	<0.001/74.0	0.92(0.79-1.07)	<0.001/57.1	**0.80(0.67-0.95)**	<0.001/59.8	**0.81(0.70-0.93)**	<0.001/70.5	**0.88(0.80-0.98)**	<0.001/74.3
Non-cancer	59 (5709/8527)	**0.77(0.68-0.88)**	<0.001/63.4	0.93(0.78-1.10)	<0.001/56.8	**0.80(0.66-0.97)**	<0.001/57.7	**0.76(0.67-0.87)**	<0.001/58.8	**0.86(0.76-0.95)**	<0.001/68.8
Sensitivity analysis											
HWE and Quality score > 15											
Overall	77 (9590/14876)	**0.76(0.68-0.85)**	<0.001/69	**0.85(0.75-0.96)**	<0.001/53.7	**0.73(0.64-0.84)**	<0.001/55	**0.78(0.70-0.88)**	<0.001/65.1	**0.83(0.77-0.90)**	<0.001/69.3
Ethnicity											
Caucasian	30 (3159/6126)	**0.81(0.68-0.96)**	<0.001/66.9	**0.85(0.73-0.98)**	0.034/34.6	**0.82(0.70-0.96)**	0.045/32.7	**0.82(0.69-0.97)**	<0.001/61.4	**0.84(0.74-0.96)**	<0.001/67.5
Asian	26 (3942/5738)	**0.74(0.61-0.90)**	<0.001/75.5	0.90(0.81-1.00)	0.021/39.4	**0.74(0.60-0.90)**	0.001/54.4	**0.75(0.61-0.93)**	<0.001/76.3	**0.83(0.75-0.93)**	<0.001/64.0
Indian	9 (1197/1244)	**0.56(0.46-0.68)**	0.085/42.4	0.80(0.47-1.37)	<0.001/86.6	**0.55(0.31-0.97)**	<0.001/80.8	**0.60(0.49-0.74)**	0.677/0	**0.73(0.53-0.99)**	<0.001/85.5
African	6 (282/241)	0.78(0.41-1.50)	0.064/52.1	0.85(0.56-1.30)	0.128/41.6	0.73(0.34-1.60)	0.067/51.4	0.86(0.55-1.33)	0.245/25.2	0.88(0.55-1.41)	0.009/67.2
Mixed	8 (1010/1527)	0.95(0.71-1.29)	0.037/53.1	**0.77(0.63-0.95)**	0.273/19.8	**0.72(0.54-0.94)**	0.143/35.8	1.00(0.81-1.24)	0.087/43.7	0.92(0.76-1.11)	0.049/50.5
Region											
Europe	23 (2280/4619)	**0.74(0.59-0.91)**	<0.001/71.4	0.90(0.75-1.08)	0.292/12.4	**0.80(0.66-0.96)**	0.073/31.8	**0.73(0.59-0.91)**	<0.001/65.6	**0.80(0.68-0.95)**	<0.001/69.5
South Asia	14 (1876/2031)	**0.69(0.59-0.80)**	0.166/26.9	0.81(0.59-1.12)	<0.001/76.3	**0.63(0.45-0.89)**	<0.001/65.5	**0.71(0.61-0.84)**	0.409/3.8	**0.79(0.67-0.94)**	<0.001/70.3
East Asia	21 (3263/4951)	**0.73(0.57-0.92)**	<0.001/79.7	0.91(0.81-1.03)	0.048/36.7	**0.75(0.59-0.94)**	0.001/57.4	**0.73(0.56-0.94)**	<0.001/80.7	**0.84(0.74-0.95)**	<0.001/67.0
Africa	8 (848/1023)	**0.73(0.57-0.94)**	0.088/43.6	**0.77(0.63-0.94)**	0.221/26.1	**0.62(0.47-0.83)**	0.123/38.5	0.79(0.60-1.03)	0.318/14.3	0.82(0.65-1.03)	0.022/57.2
South America	6 (606/1154)	1.18(0.94-1.48)	0.335/12.6	0.84(0.59-1.18)	0.082/48.9	0.99(0.68-1.44)	0.219/28.8	1.22(0.96-1.55)	0.236/26.4	1.05(0.89-1.24)	0.197/31.8
North America	5 (717/1098)	0.87(0.53-1.40)	0.003/75.1	0.78(0.31-1.98)	<0.001/85.9	0.76(0.28-2.05)	<0.001/85.2	0.99(0.80-1.21)	0.471/0	0.82(0.47-1.42)	<0.001/90.1
Matching											
YES	47 (6521/9613)	**0.74(0.64-0.85)**	<0.001/68.3	**0.82(0.70-0.97)**	<0.001/63.8	**0.68(0.56-0.81)**	<0.001/59	**0.76(0.66-0.88)**	<0.001/65.2	**0.83(0.75-0.91)**	<0.001/69.2
NR	30 (3069/5263)	**0.80(0.65-0.97)**	<0.001/70.8	0.87(0.76-1.01)	0.151/21.2	**0.82(0.70-0.96)**	0.006/43.7	**0.81(0.67-0.98)**	<0.001/65.8	**0.84(0.73-0.96)**	<0.001/70.3
Source of controls											
Healthy	40 (5457/9147)	**0.75(0.64-0.88)**	<0.001/74.1	0.84(0.71-1.10)	<0.001/58.5	**0.72(0.59-0.87)**	<0.001/59.3	**0.78(0.65-0.91)**	<0.001/70.8	**0.83(0.75-0.92)**	<0.001/72.8
Non-cancer	37 (4133/5729)	**0.78(0.66-0.91)**	<0.001/61.7	**0.85(0.76-0.95)**	<0.001/48.6	**0.75(0.61-0.92)**	<0.001/50.6	**0.80(0.68-0.93)**	<0.001/57.1	**0.84(0.75-0.94)**	<0.001/65.6

*p53 rs1042522:* allele model: Pro vs. Arg, homozygous model: Pro/Pro vs. Arg/Arg, dominant model: Pro/Pro + Arg/Pro vs. Arg/Arg, recessive model: Pro/Pro vs. Arg/Arg+ Arg/Pro, Codominance model: Arg/Pro vs. Arg/Arg; HWE, Hardy–Weinberg Equilibrium; NR, Not Reported.

Bold type represents a positive result of the study.

#### *p53 rs17878362* polymorphism and cervical cancer

**Figure 4:**
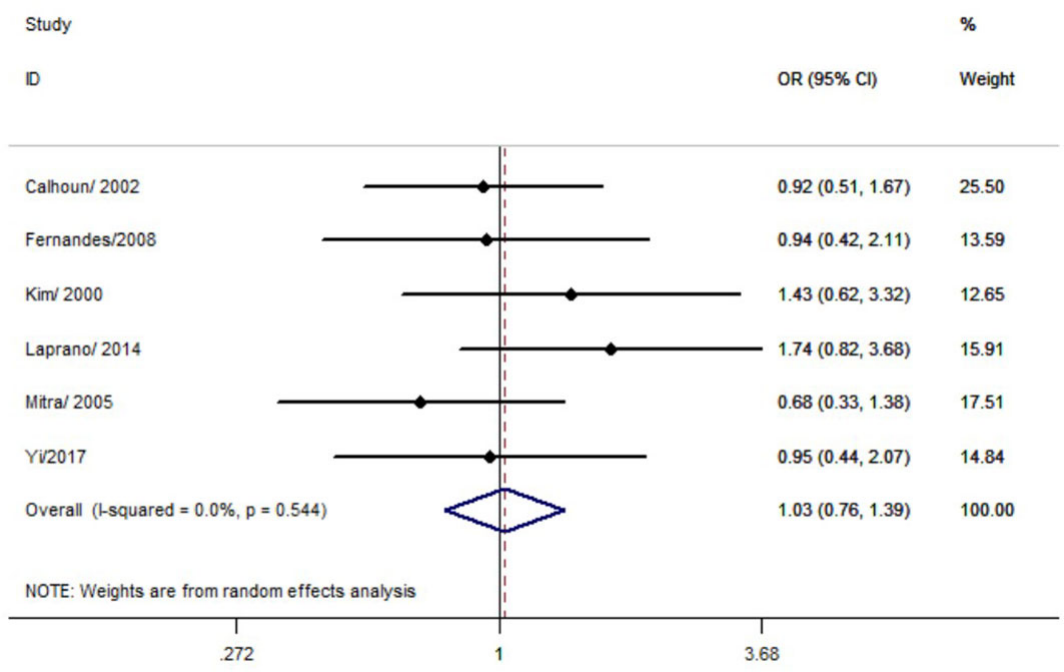
Forest map of the correlation between *p53 rs17878362* polymorphism and cervical cancer in overall analysis (A2/A2+ A1/A2 vs. A1/A1).

**Figure 5:**
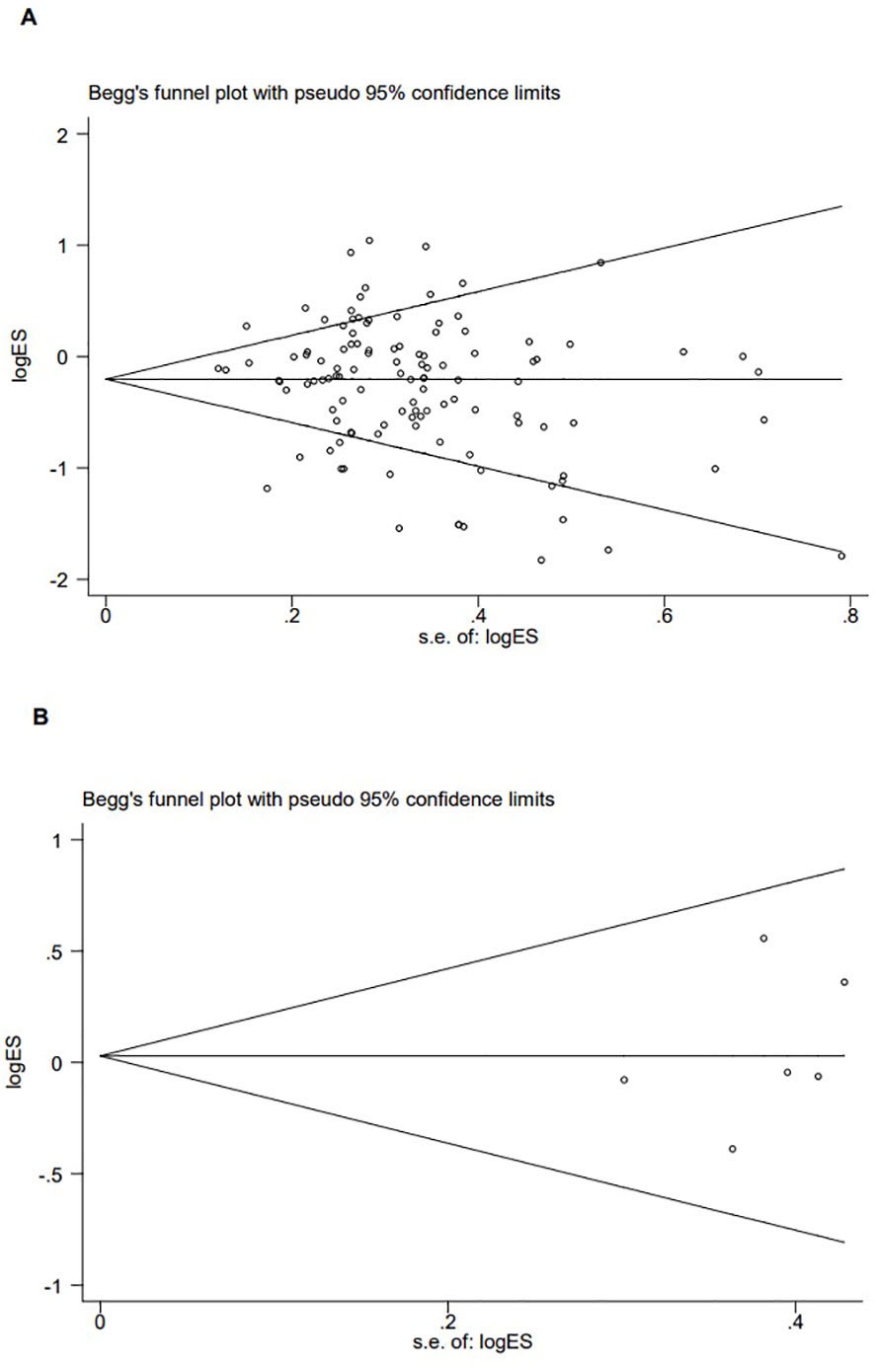
Publication bias of the combined effect of Begg funnel plot assessment of *p53 rs1042522* [**(A)** Pro Pro +Arg Pro vs. Arg Arg) and *rs17878362* [**(B)** Pro Pro +Arg Pro vs. Arg Arg) polymorphisms and cervical cancer.

No significant association was observed between the *p53* rs17878362 polymorphism and risk of cervical cancer in the overall population (**Table 2**, **Figure 4**). Sensitivity analysis revealed consistent results without significant changes. Additionally, no publication bias was detected based on Egger’s test and Begg’s funnel plot (A2/A2+ A1/A2 vs. A1/A1: *P* = 0.48; A2/A2 vs. A1/A1+ A1/A2: *P* = 0.59; A2/A2 vs. A1/A1: *P* = 0.60; A1/A2 vs. A1/A1: p=0.48; A1 vs. A2: *P* = 0.65). In the overall analysis, the results for the Pro Pro +Arg Pro vs. Arg Arg models did not change (data not shown), suggesting that more studies could not change the pooled results (**Figure 5**).

**Table 2:** Meta-analysis of the association of *p53 rs17878362* polymorphism with risk of cervical cancer.

Variable	n (Cases/Controls)	A2/A2+ A1/A2 *vs.* A1/A1		A2/A2 *vs.* A1/A1+ A1/A2		A2/A2 *vs.* A1/A1		A1/A2 *vs*. A1/A1		A2 *vs.* A1	
		OR (95% CI)	*P_h_/I^2^* (%)	OR (95% CI)	*P_h_/I^2^* (%)	OR (95% CI)	*P_h_/I^2^* (%)	OR (95% CI)	*P_h_/I^2^* (%)	OR (95% CI)	*P_h_/I^2^* (%)
Overall	6 (664/687)	1.03(0.76-1.38)	0.544/0	0.95(0.26-3.43)	0.64/0	0.97(0.27-3.49)	0.533/0	1.03(0.76-1.40)	0.634/0	1.11(0.84-1.46)	0.54/0
Ethnicity											
Caucasian	2 (158/133)	0.98(0.58-1.66)	0.668/0	2.51(0.10-64.27)	NA	2.58(0.10-67.27)	NA	0.96(0.57-1.62)	0.791/0	1.01(0.62-1.63)	0.556/0
Asian	2 (348/341)	1.15(0.65-2.03)	0.485/0	NA	NA	NA	NA	1.15(0.65-2.03)	0.485/0	1.15(0.66-2.00)	0.492/0
Mixed	2 (97/120)	1.38(0.73-2.59)	0.261/20.9	0.95(0.19-4.65)	0.232/30.1	1.03(0.21-5.10)	0.197/39.8	1.43(0.74-2.74)	0.43/0	1.24(0.46-3.30)	0.111/60.7
Indian	1 (61/93)	–	–	–	–	–	–	–	–	–	–
Matching											
YES	4 (452/339)	0.93(0.65-1.32)	0.620/0	0.56(0.10-3.25)	0.865/0	0.53(0.09-3.04)	0.826/0	0.94(0.66-1.35)	0.661/0	1.00(0.72-1.40)	0.790/0
NR	2 (212/248)	1.30(0.76-2.22)	0.273/16.8	2.0(0.27-14.69)	NA	2.39(0.32-17.85)	NA	1.27(0.73-2.19)	0.306/4.5	1.40(0.85-2.29)	0.186/42.9
Source of controls											
Healthy controls	3 (431/398)	1.08(0.68-1.71)	0.721/0	0.68(0.04-11.15)	NA	0.68(0.04-11.11)	NA	1.09(0.68-1.74)	0.736/0	1.12(0.72-1.76)	0.783/0
Non-cancer controls	3 (233/289)	0.99(0.70-1.46)	0.189/40	1.03(0.25-4.36)	0.369/0	1.06(0.25-4.47)	0.283/13.3	0.99(0.67-1.47)	0.253/27.2	1.10(0.78-1.57)	0.167/44
Sensitivity analysis											
HWE and Quality score > 15											
Overall	5 (619/599)	0.93(0.68-1.28)	0.776/0	0.56(0.10-3.25)	0.865/0	0.53(0.09-3.04)	0.826/0	0.95(0.68-1.31)	0.81/0	0.99(0.73-1.35)	0.901/0

*p53 rs17878362*: allele model: A2 vs. A1, homozygous model: A2/A2 vs. A1/A1, dominant model: A2/A2+ A1/A2 vs. A1/A1, recessive model: A2/A2 vs. A1/A1+ A1/A2, Codominance model: A1/A2 *vs*. A1/A1; HWE, Hardy–Weinberg Equilibrium; NR, Not Reported.

NA, Not available.

### Credibility analysis

In our study, the credibility of all significant associations was evaluated using FPRP, BFDP, and Venice criteria; however, they were deemed as having lower credibility (**Table 3**).

**Table 3:** FPRP and BFDP of the current meta-analysis.

Gene	Variable	Model	N/sample size	SMD	P_h_/I^2^ (%)	False Discovery Rate			
						Prior probability of 0.001			
						Power	FPRP		BFDP
*72*	Overall	**Pro/Pro +Arg/Pro *vs.* Arg/Arg**	114 (12655/19272)	**0.79 (0.71-0.87)**	<0.001/69.7	0.139	0.012		0.115
*72*	Overall	**Pro/Pro *vs.* Arg/Arg**	114 (12655/19272)	**0.80 (0.70-0.91)**	<0.001/58.4	0.267	0.720		0.967
*72*	Overall	**Arg/Pro *vs*. Arg/Arg**	114 (12655/19272)	**0.78 (0.71-0.86)**	<0.001/65.2	0.092	0.007		0.938
*72*	Overall	**Pro *vs.* Arg**	114 (12655/19272)	**0.87 (0.81-0.93)**	<0.001/71.5	0.897	0.045		0.797
*72*	Caucasian	**Pro/Pro +Arg/Pro *vs.* Arg/Arg**	40 (4020/7676)	**0.81 (0.70-0.94)**	<0.001/62.2	0.354	0.940		0.994
*72*	Caucasian	**Pro/Pro *vs.* Arg/Arg**	40 (4020/7676)	**0.84 (0.73-0.98)**	0.063/26.9	0.540	0.980		0.998
*72*	Caucasian	**Arg/Pro *vs*. Arg/Arg**	40 (4020/7676)	**0.81 (0.70-0.94)**	<0.001/57.4	0.354	0.940		0.994
*72*	Caucasian	**Pro *vs.* Arg**	40 (4020/7676)	**0.86 (0.77-0.96)**	<0.001/61.5	0.713	0.910		0.966
*72*	Asian	**Pro/Pro +Arg/Pro *vs.* Arg/Arg**	44 (5663/7610)	**0.80 (0.67-0.95)**	<0.001/78.2	0.321	0.971		0.996
*72*	Asian	**Arg/Pro *vs*. Arg/Arg**	44 (5663/7610)	**0.78 (0.66-0.93)**	<0.001/75	0.231	0.961		0.993
*72*	Asian	**Pro *vs.* Arg**	44 (5663/7610)	**0.89 (0.79-0.99)**	<0.001/77.3	0.887	0.973		0.999
*72*	Indian	**Pro/Pro +Arg/Pro *vs.* Arg/Arg**	10 (1227/1924)	**0.57 (0.47-0.70)**	0.085/41	0.001	0.360		0.005
*72*	Indian	**Arg/Pro *vs*. Arg/Arg**	10 (1227/1924)	**0.60 (0.48-0.73)**	0.756/0	0.001	0.391		0.018
*72*	Mixed	**Pro/Pro *vs.* Arg/Pro + Arg/Arg**	14 (1378/2314)	**0.81 (0.68-0.98)**	0.248/18.9	0.385	0.987		0.998
*72*	Mixed	**Pro/Pro *vs.* Arg/Arg**	14 (1378/2314)	**0.73 (0.57-0.92)**	0.480/0	0.131	0.983		0.994
*72*	Mixed	**Pro *vs.* Arg**	14 (1378/2314)	**0.88 (0.79-0.98)**	0.090/35.7	0.839	0.960		0.999
*72*	Europe	**Pro/Pro +Arg/Pro *vs.* Arg/Arg**	32 (3118/6007)	**0.77 (0.65-0.92)**	<0.001/65.3	0.192	0.954		0.991
*72*	Europe	**Pro/Pro *vs.* Arg/Arg**	32 (3118/6007)	**0.84 (0.70-0.99)**	0.09/26.2	0.538	0.986		0.999
*72*	Europe	**Arg/Pro *vs*. Arg/Arg**	32 (3118/6007)	**0.76 (0.64-0.91)**	<0.001/60	0.158	0.947		0.988
*72*	Europe	**Pro *vs.* Arg**	32 (3118/6007)	**0.84 (0.74-0.96)**	<0.001/62.9	0.547	0.950		0.997
*72*	East Asia	**Pro/Pro +Arg/Pro *vs.* Arg/Arg**	36 (4671/6544)	**0.74 (0.61-0.90)**	<0.001/77.2	0.117	0.956		0.986
*72*	East Asia	**Pro/Pro *vs.* Arg/Arg**	36 (4671/6544)	**0.76 (0.62-0.94)**	<0.001/63.1	0.198	0.983		0.996
*72*	East Asia	**Arg/Pro *vs*. Arg/Arg**	36 (4671/6544)	**0.72 (0.59-0.88)**	<0.001/76.5	0.077	0.946		0.974
*72*	East Asia	**Pro *vs.* Arg**	36 (4671/6544)	**0.84 (0.75-0.95)**	<0.001/72.4	0.550	0.909		0.995
*72*	Africa	**Pro/Pro +Arg/Pro *vs.* Arg/Arg**	10 (933/1160)	**0.75 (0.59-0.95)**	0.174/29.4	0.191	0.989		0.997
*72*	Africa	**Pro/Pro *vs.* Arg/Arg**	10 (933/1160)	**0.69 (0.48-0.98)**	0.165/30.5	0.146	0.996		0.998
*72*	YES	**Pro/Pro +Arg/Pro *vs.* Arg/Arg**	58 (7490/10883)	**0.78 (0.68-0.90)**	<0.001/73.9	0.182	0.785		0.963
*72*	YES	**Pro/Pro *vs.* Arg/Arg**	58 (7490/10883)	**0.75 (0.63-0.90)**	<0.001/64.2	0.129	0.939		0.983
*72*	YES	**Arg/Pro *vs*. Arg/Arg**	58 (7490/10883)	**0.79 (0.68-0.91)**	<0.001/70.9	0.230	0.825		0.963
*72*	YES	**Pro *vs.* Arg**	58 (7490/10883)	**0.88 (0.80-0.97)**	<0.001/74.3	0.864	0.921		0.998
*72*	NR	**Pro/Pro +Arg/Pro *vs.* Arg/Arg**	56 (5165/8389)	**0.79 (0.68-0.91)**	<0.001/64.3	0.230	0.825		0.976
*72*	NR	**Pro/Pro *vs.* Arg/Arg**	56 (5165/8389)	**0.83 (0.74-0.94)**	<0.001/49.8	0.475	0.875		0.992
*72*	NR	**Arg/Pro *vs*. Arg/Arg**	56 (5165/8389)	**0.78 (0.68-0.90)**	<0.001/57.4	0.182	0.785		0.963
*72*	NR	**Pro *vs.* Arg**	56 (5165/8389)	**0.86 (0.77-0.96)**	<0.001/68.3	0.713	0.910		0.996
*72*	Healthy	**Pro/Pro +Arg/Pro *vs.* Arg/Arg**	55 (6946/10745)	**0.80 (0.69-0.92)**	<0.001/74	0.283	0.861		0.985
*72*	Healthy	**Pro/Pro *vs.* Arg/Arg**	55 (6946/10745)	**0.80 (0.67-0.95)**	<0.001/59.8	0.321	0.971		0.996
*72*	Healthy	**Arg/Pro *vs*. Arg/Arg**	55 (6946/10745)	**0.81 (0.70-0.93)**	<0.001/70.5	0.344	0.890		0.990
*72*	Healthy	**Pro *vs.* Arg**	55 (6946/10745)	**0.88 (0.80-0.98)**	<0.001/74.3	0.839	0.960		0.999
*72*	Non-cancer	**Pro/Pro +Arg/Pro *vs.* Arg/Arg**	59 (5709/8527)	**0.77 (0.68-0.88)**	<0.001/63.4	0.123	0.504		0.854
*72*	Non-cancer	**Pro/Pro *vs.* Arg/Arg**	59 (5709/8527)	**0.80 (0.66-0.97)**	<0.001/57.7	0.339	0.986		0.998
*72*	Non-cancer	**Arg/Pro *vs*. Arg/Arg**	59 (5709/8527)	**0.76 (0.67-0.87)**	<0.001/58.8	0.091	0.432		0.770
*72*	Non-cancer	**Pro *vs.* Arg**	60 (5749/8547)	**0.86 (0.76-0.95)**	<0.001/68.8	0.732	0.802		0.993
Sensitivity analysis HWE and Quality score > 15									
72	Overall	**Pro/Pro + Arg/Pro *vs.* Arg/Arg**	77 (9590/14876)	**0.76 (0.68-0.85)**	<0.001/69	0.053	0.028		0.097
72	Overall	**Pro/Pro *vs.* Arg/Pro + Arg/Arg**	77 (9590/14876)	**0.85 (0.75-0.96)**	<0.001/53.7	0.625	0.934		0.997
72	Overall	**Pro/Pro *vs.* Arg/Arg**	77 (9590/14876)	**0.73 (0.64-0.84)**	<0.001/55	0.032	0.256		0.371
72	Overall	**Arg/Pro *vs*. Arg/Arg**	77 (9590/14876)	**0.78 (0.70-0.88)**	<0.001/65.1	0.141	0.277		0.745
72	Overall	**Pro *vs.* Arg**	77 (9590/14876)	**0.83 (0.77-0.90)**	<0.001/69.3	0.461	0.014		0.354
72	Caucasian	**Pro/Pro +Arg/Pro *vs.* Arg/Arg**	30 (3159/6126)	**0.81 (0.68-0.96)**	<0.001/66.9	0.372	0.976		0.997
72	Caucasian	**Pro/Pro *vs.* Arg/Pro + Arg/Arg**	30 (3159/6126)	**0.85 (0.73-0.98)**	0.034/34.6	0.607	0.976		0.998
72	Caucasian	**Pro/Pro *vs.* Arg/Arg**	30 (3159/6126)	**0.82 (0.70-0.96)**	0.045/32.7	0.421	0.970		0.997
72	Caucasian	**Arg/Pro *vs*. Arg/Arg**	30 (3159/6126)	**0.82 (0.69-0.97)**	<0.001/61.4	0.425	0.980		0.998
72	Caucasian	**Pro *vs.* Arg**	30 (3159/6126)	**0.84 (0.74-0.96)**	<0.001/67.5	0.547	0.950		0.997
72	Asian	**Pro/Pro +Arg/Pro *vs.* Arg/Arg**	26 (3942/5738)	**0.74 (0.61-0.90)**	<0.001/75.5	0.117	0.956		0.986
72	Asian	**Pro/Pro *vs.* Arg/Arg**	26 (3942/5738)	**0.74 (0.60-0.90)**	0.001/54.4	0.117	0.956		0.986
72	Asian	**Arg/Pro *vs*. Arg/Arg**	26 (3942/5738)	**0.75 (0.61-0.93)**	<0.001/76.3	0.169	0.981		0.995
72	Asian	**Pro *vs.* Arg**	26 (3942/5738)	**0.83 (0.75-0.93)**	<0.001/64.0	0.472	0.737		0.983
72	Indian	**Pro/Pro +Arg/Pro *vs.* Arg/Arg**	9 (1197/1244)	**0.56 (0.46-0.68)**	0.085/42.4	0.001	0.138		0.001
72	Indian	**Pro/Pro *vs.* Arg/Arg**	9 (1197/1244)	**0.55 (0.31-0.97)**	<0.001/80.8	0.076	0.998		0.998
72	Indian	**Arg/Pro *vs*. Arg/Arg**	9 (1197/1244)	**0.60 (0.49-0.74)**	0.677/0	0.001	0.391		0.018
72	Indian	**Pro *vs.* Arg**	9 (1197/1244)	**0.73 (0.53-0.99)**	<0.001/85.5	0.197	0.995		0.998
72	Mixed	**Pro/Pro *vs.* Arg/Pro + Arg/Arg**	8 (1010/1527)	**0.77 (0.63-0.95)**	0.273/19.8	0.230	0.985		0.997
72	Mixed	**Pro/Pro *vs.* Arg/Arg**	8 (1010/1527)	**0.72 (0.54-0.94)**	0.143/35.8	0.141	0.991		0.996
72	Europe	**Pro/Pro + Arg/Pro *vs.* Arg/Arg**	23 (2280/4619)	**0.74 (0.59-0.91)**	<0.001/71.4	0.130	0.971		0.991
72	Europe	**Pro/Pro *vs.* Arg/Arg**	23 (2280/4619)	**0.80 (0.66-0.96)**	0.073/31.8	0.267	0.720		0.967
72	Europe	**Arg/Pro *vs*. Arg/Arg**	23 (2280/4619)	**0.73 (0.59-0.91)**	<0.001/65.6	0.120	0.977		0.992
72	Europe	**Pro *vs.* Arg**	23 (2280/4619)	**0.80 (0.68-0.95)**	<0.001/69.5	0.321	0.971		0.996
72	South Asia	**Pro/Pro +Arg/Pro *vs.* Arg/Arg**	14 (1876/2031)	**0.69 (0.59-0.80)**	0.166/26.9	0.013	0.975		0.932
72	South Asia	**Pro/Pro *vs.* Arg/Arg**	14 (1876/2031)	**0.63 (0.45-0.89)**	<0.001/65.5	0.056	0.994		0.993
72	South Asia	**Arg/Pro *vs*. Arg/Arg**	14 (1876/2031)	**0.71 (0.61-0.84)**	0.409/3.8	0.031	0.679		0.733
72	South Asia	**Pro *vs.* Arg**	14 (1876/2031)	**0.79 (0.67-0.94)**	<0.001/70.3	0.274	0.966		0.995
72	East Asia	**Pro/Pro +Arg/Pro *vs.* Arg/Arg**	21 (3263/4951)	**0.73 (0.57-0.92)**	<0.001/79.7	0.131	0.983		0.994
72	East Asia	**Pro/Pro *vs.* Arg/Arg**	21 (3263/4951)	**0.75 (0.59-0.94)**	0.001/57.4	0.180	0.986		0.996
72	East Asia	**Arg/Pro *vs*. Arg/Arg**	21 (3263/4951)	**0.73 (0.56-0.94)**	<0.001/80.7	0.152	0.990		0.996
72	East Asia	**Pro *vs.* Arg**	21 (3263/4951)	**0.84 (0.74-0.95)**	<0.001/67.0	0.550	0.909		0.995
72	YES	**Pro/Pro +Arg/Pro *vs.* Arg/Arg**	47 (6521/9613)	**0.74 (0.64-0.85)**	<0.001/68.3	0.046	0.307		0.515
72	YES	**Pro/Pro *vs.* Arg/Pro + Arg/Arg**	47 (6521/9613)	**0.82 (0.70-0.97)**	<0.001/63.8	0.425	0.980		0.998
72	YES	**Pro/Pro *vs.* Arg/Arg**	47 (6521/9613)	**0.82 (0.70-0.97)**	<0.001/63.8	0.425	0.980		0.998
72	YES	**Arg/Pro *vs*. Arg/Arg**	47 (6521/9613)	**0.76 (0.66-0.88)**	<0.001/65.2	0.109	0.690		0.910
72	YES	**Pro *vs.* Arg**	47 (6521/9613)	**0.83 (0.75-0.91)**	<0.001/69.2	0.466	0.134		0.828
72	NR	**Pro/Pro +Arg/Pro *vs.* Arg/Arg**	30 (3069/5263)	**0.80 (0.65-0.97)**	<0.001/70.8	0.339	0.986		0.998
72	NR	**Pro/Pro *vs.* Arg/Arg**	30 (3069/5263)	**0.82 (0.70-0.96)**	0.006/43.7	0.421	0.970		0.997
72	NR	**Arg/Pro *vs*. Arg/Arg**	30 (3069/5263)	**0.81 (0.67-0.98)**	<0.001/65.8	0.385	0.987		0.998
72	NR	**Pro *vs.* Arg**	30 (3069/5263)	**0.84 (0.73-0.96)**	<0.001/70.3	0.547	0.950		0.997
72	Healthy	**Pro/Pro +Arg/Pro *vs.* Arg/Arg**	40 (5457/9147)	**0.75 (0.64-0.88)**	<0.001/74.1	0.098	0.810		0.940
72	Healthy	**Pro/Pro *vs.* Arg/Arg**	40 (5457/9147)	**0.72 (0.59-0.87)**	<0.001/59.3	0.061	0.940		0.966
72	Healthy	**Arg/Pro *vs*. Arg/Arg**	40 (5457/9147)	**0.78 (0.65-0.91)**	<0.001/70.8	0.200	0.888		0.985
72	Healthy	**Pro *vs.* Arg**	40 (5457/9147)	**0.83 (0.75-0.92)**	<0.001/72.8	0.470	0.453		0.954
72	Non-cancer	**Pro/Pro +Arg/Pro *vs.* Arg/Arg**	37 (4133/5729)	**0.78 (0.66-0.91)**	<0.001/61.7	0.200	0.888	0.982	
72	Non-cancer	**Pro/Pro *vs.* Arg/Pro + Arg/Arg**	37 (4133/5729)	**0.85 (0.76-0.95)**	<0.001/48.6	0.636	0.868	0.994	
72	Non-cancer	**Pro/Pro *vs.* Arg/Arg**	37 (4133/5729)	**0.75 (0.61-0.92)**	<0.001/50.6	0.156	0.974	0.993	
72	Non-cancer	**Arg/Pro *vs*. Arg/Arg**	37 (4133/5729)	**0.80 (0.68-0.93)**	<0.001/57.1	0.298	0.925	0.992	
72	Non-cancer	**Pro *vs.* Arg**	37 (4133/5729)	**0.84 (0.75-0.94)**	<0.001/65.6	0.555	0.811	0.990	

Bold type represents a positive result of the study.

## Discussion

This meta-analysis comprised a total of 125 studies from 114 articles. The application of genetic models in meta-analysis can help us to better reveal the true association between genes and diseases, based on previous research, we chose five genetic models (dominant model; recessive model; homozygous model; codominance model; allele model). Moreover, excluding low-quality studies would provide a more accurate representation of this relationship. Additionally, our findings indicated that *p53* rs1042522 polymorphism significantly influenced cervical cancer risk in both matched and control subgroups, suggesting that matching factors and control variables did not affect its association with cervical cancer. However, after considering the reliability of the results, this study indicates that the p53 rs1042522 polymorphism is not associated with the cervical cancer risk. Furthermore, no significant association was found between the *p53* rs17878362 polymorphism and cervical cancer risk, these results were consistent with those obtained from sensitivity analysis.

It is important to note that meta-analysis of gene polymorphisms involves aggregation of extensive genomic data which may lead to false positive results; therefor credibility assessment using FPRP, BFDP, and Venice criteria is commonly employed. Based on analytical evaluation using these criteria, we concluded that the confidence intervals for the associations between *p53* rs1042522 polymorphism with cervical cancer risk were relatively unreliable. Up to now, a total of nine meta-analyses have investigated the association between *p53* rs1042522 polymorphism and the risk of cervical cancer. Francisco et al. (7) and Yu et (14) al found that the *p53* rs1042522 was correlated with an increased risk of cervical cancer in whole population. Koushik et al. (11) found was same conclusion, but the number of deviations from Hardy-Weinberg equilibrium in the control group of the included studies was large, which led to an inevitable decrease in the reliability of the conclusions. Kamiza et al. (9) and Li et al. (12) observed that the p53 rs1042522 was associated with an increased risk of cervical cancer in Africans and Chinese population, respectively. Zhou et al. (15) study also found the same results in Asians. Habbous et al. (8) found that the Arg variant is associated with progression of Squamous Intraepithelial Lesion to cervical cancer only in the presence of Human Papillomavirus positivity. Sousa et al. (13) found that p53 codon 72 polymorphism in countries with low incidence rates of cervical cancer, this polymorphism might represent a significant genetic marker. Hower, Klug et al. (10) found that the p53 rs1042522 was not association with risk of cervical cancer. Inconsistencies in the existence of previous studies may be due to differences in the number of studies included in the studies and differences in the study populations. The cases and controls of Klug et al. (10) study most were white women, this can lead to pooling bias. There exist contradictory conclusions among these studies. Moreover, some articles with weak associations were included in the meta-analysis without strict evaluation of their quality. Additionally, none of them accounted for potential false positive results.

To address these conflicting conclusions and determine the precise association between *p53* rs1042522 and *p53* rs17878362 with cervical cancer, an updated meta-analysis is deemed necessary. The strengths of this updated meta-analysis are as follows: (1) It includes a larger sample size comprising 114 articles compared to previous studies; (2) HWE was assessed in control group; (3) Credibility evaluation was conducted on significant results; (4) Ethnic differences were thoroughly analyzed. However, our study also has certain limitations. Firstly, we only considered eligible studies from specific databases without exploring alternative sources for eligible studies. Secondly, our search was limited to English and Chinese languages while excluding articles published in other languages. Lastly, the genotype data we included were unadjusted. Because of study limitations, we did not adjust for miscarriage, presence or absence of HPV infection, and other factors. Hence, future research should aim to include more comprehensive adjustments for confounding factors in order to obtain accurate conclusions.

## Conclusion

In conclusion, the significant association between *p53* rs1042522 polymorphism and the risk of cervical cancer may be false positive results. More research is needed to confirm this association.

## Data Availability

The original contributions presented in the study are included in the article/**Supplementary Material**. Further inquiries can be directed to the corresponding author/s.
