# Supplementary material for: Association between the p53 polymorphisms and cervical cancer risk: an updated meta-analysis
Source: Front Oncol. 2025 Feb 21;15:1461737. doi: 10.3389/fonc.2025.1461737 (PMC11885137; doi:10.3389/fonc.2025.1461737)

**Supplemental Table 1** Scale for quality assessment of molecular association studies of CC

Criterion

Score

Source of case

Selected from population or cancer registry

Selected from hospital

3

2

[1](#br1)

[0](#br1)

Selected from pathology archives, but without description

Not described

Source of control

Population-based

3

2

[1](#br1)

[0](#br1)

Blood donors or volunteers

Hospital-based

Not described

Ascertainment of cancer

Histological or pathological confirmation

Diagnosis of BC by patient medical record

Not described

2

[1](#br1)

[0](#br1)

Ascertainment of control

Controls were tested to screen out BC

Controls were subjects who did not report BC, no objective testing

Not described

2

[1](#br1)

[0](#br1)

Matching

Controls matched with cases by age

Not matched or not described

Source of genotyping material of case

Appropriate DNA sources (such as peripheral blood, buccal swabs ,exfoliated cells and so on)

2

[0](#br1)

2

[1](#br1)

[0](#br1)

Tumor tissue

Not described

Genotyping examination

Genotyping done blindly and quality control

2

[1](#br1)

[0](#br1)

Only genotyping done blindly or quality control

Unblinded and without quality control

HWE

HWE in the control group

2

[0](#br1)

Hardy-Weinberg disequilibrium in the control group

Association assessment

Assess association between genotypes and CC with appropriate statistics and adjustment for confounders

2

[1](#br1)

[0](#br1)

Assess association between genotypes and CC with appropriate statistics without adjustment for confounders

Inappropriate statistics used

Total sample size

＞500

3

2

1

200-500

<200

HWE: Hardy-Weinberg equilibrium, CC: cervical cancer


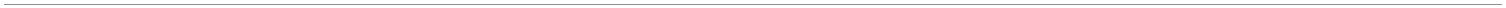

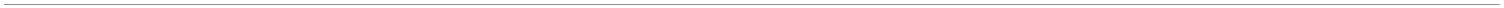

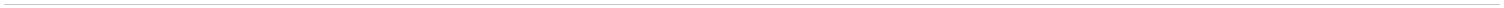

Supplement: Supplementary file 1 [file DataSheet1.zip › Supplementary Table 1.DOCX]
