# Supplementary material for: Association between the p53 polymorphisms and cervical cancer risk: an updated meta-analysis
Source: Front Oncol. 2025 Feb 21;15:1461737. doi: 10.3389/fonc.2025.1461737 (PMC11885137; doi:10.3389/fonc.2025.1461737)
Supplement: Supplementary file 1 [file DataSheet1.zip › Supplementary Table 2.DOCX]

| **S2 Table. Characteristics of the studies of *P53 rs1042522* polymorphism included in the meta-analysis (A, Asian; I: Indian; Af, African; C, Caucasian; M, Mixed; U, Unidentified; NA,Not Available; HWD, Hardy-Weinberg Disequilibrium).** | | | | | | | | | | | |
| --- | --- | --- | --- | --- | --- | --- | --- | --- | --- | --- | --- |
| First Author/ Year | Country | **All** **studies** | | | | | | | | | |
|  |  | This study, 2023 | Yu [7]2022 | Kamiza [8]2020 | Li [9]2015 | Habbous [10]2012 | Zhou [11] 2012 | Francisco [12] 2010 | Klug [13]2009 | Sousa [14]2007 | Koushik [15] 2004 |
| Abba/ 2003 | Argentina | M |  |  |  |  |  | M | M |  |  |
| Agorastos/ 2000 | Greek | C |  |  |  | C (NA) |  | C | C | C (NA) | C |
| Alsbeih/ 2013 | Saudi Arabia | A | A |  |  |  |  |  |  |  |  |
| Andersson/ 2001 | Sweden | C |  |  |  | C (NA) |  | C |  |  | C |
| Apu/ 2020 | Bangladesh | A(HWD) | A |  |  |  |  |  |  |  |  |
| Arbel-Alon/ 2002 | Israel | C(HWD) |  |  |  |  |  | C (HWD) | C (HWD) |  | C (HWD) |
| Assoumou/ 2015 | Gabon | Af | Af | Af (NA) |  |  |  |  |  |  |  |
| Baek/ 2000 | Korea | A |  |  |  | A (NA) | A (NA) | A (HWD) |  |  | A (HWD) |
| Barbisan / 2011 | Argentina | M(HWD) | M |  |  |  |  |  |  |  |  |
| Bertorelle/ 1999 | Italy | C |  |  |  | C (NA) |  | C | C | C (NA) | C |
| Bhattacharya/ 2002 | India | I |  |  |  |  | I (NA) | I | I |  | I |
| Bhattacharya/ 2005 | India | I |  |  |  | I (NA) |  | I |  |  |  |
| Boumba/ 2017 | Congo | Af(HWD) |  | Af (NA) |  |  |  |  |  |  |  |
| Brady/ 1999 | UK | C |  |  |  | C (NA) |  | C |  | C (NA) | C |
| Calhoun/ 2002 | USA | C |  |  |  |  |  | C |  |  | C |
| Cenci/ 2003 | Italy | C(HWD) |  |  |  |  |  | C |  | C (NA) |  |
| Chen/ 2012 | China | A |  |  | A |  |  |  |  |  |  |
| Cho/ 2003 | Korea | A |  |  |  | A (NA) | A (NA) |  |  |  |  |
| Ciotti/ 2006 | Italy | C |  |  |  |  |  |  | C |  |  |
| Comar/ 2003 | Italy | C |  |  |  | C (NA) |  | C |  | C (NA) |  |
| Datkhile/ 2019 | India |  | I (NA) |  |  |  |  |  |  |  |  |
| Dokianakis/ 2000 | Greece | C |  |  |  | C (NA) |  | C |  | C (NA) | C (HWD) |
| Dybikowska/ 2000 | Poland | C |  |  |  | C (NA) |  | C | C | C (NA) | C |
| El Khair/ 2009 | Moroccan | C |  |  |  | C (NA) |  | C |  |  |  |
| Eltahir/ 2012 | Sudan | Af |  | Af (NA) |  |  |  |  |  |  |  |
| Giannoudis/ 1999 | UK | C |  |  |  | C (NA) |  | C | C | C (NA) | C |
| González Herrera/ 2014 | Mexico | M | M |  |  |  |  |  |  |  |  |
| Govan/ 2007 | South Africa | Af |  | Af (NA) |  |  |  | Af |  |  |  |
| Gudleviciene/ 2006 | Lithuania | C | C (NA) |  |  | C (NA) |  | C (HWD) | C (HWD) |  |  |
| Gustafsson/ 2001 | Sweden | C |  |  |  | C (NA) |  | C |  | C (NA) | C |
| Hayes/ 1998 | Netherlands | C |  |  |  |  |  | C | C | C (NA) | C |
| Helland/ 1998 | Norway | C(HWD) |  |  |  | C (NA) |  | C | C | C (NA) | C |
| Hildesheim/ 1998 | Porto Rico | C |  |  |  |  |  | C | C |  | C |
| Hou/ 2006 | China | A |  |  | A |  | A (NA) |  |  |  |  |
| Humbey/ 2002 | France | C(HWD) |  |  |  |  |  |  | C (HWD) | C (NA) | C (HWD) |
| Jiang/ 2001 | China | C(HWD) |  |  | A |  |  | A |  |  |  |
| Jiang/ 2010 | China | A | A |  |  |  |  |  |  |  |  |
| Josefsson/ 1998 | Sweden | C |  |  |  | C (NA) |  | C | C (HWD) | C (NA) | C (HWD) |
| Katiyar/ 2003 | India | I |  |  |  | I (NA) | I (NA) | I |  |  |  |
| Kawamata/ 2002 | Japan | A |  |  |  |  | A (NA) | A | A |  | A |
| Kim/ 2000 | Korea | A(HWD) |  |  |  |  | A (NA) | A |  |  | A |
| Kim/ 2001 | Korea | A |  |  |  | A (NA) | A (NA) | A | A |  | A |
| Klaes/ 1999 | Germany | C |  |  |  | C (NA) |  | C | C | C (NA) | C |
| Klug/ 2001 | Peru | M | M |  |  | M |  | M | M |  | M |
| Kouamou/ 2016 | Zimbabwe | Af |  | Af (NA) |  |  |  |  |  |  |  |
| Koushik/ 2005 | Canada (Montréal) | M |  |  |  | M (NA) |  |  |  |  |  |
| Lanham/ 1998 | UK | C |  |  |  | C (NA) |  | C |  |  | C |
| Laprano/ 2014 | Brazil | M | M |  |  |  |  |  |  |  |  |
| Lee/ 2004 | Korea | A | A (NA) | A ( NA) |  |  |  | A |  |  |  |
| Lee/ 2004 | Korea | A |  |  |  |  |  | A |  |  |  |
| Li/ 2004 | China | A(HWD) |  |  | A (HWD) |  | A (NA) |  |  |  |  |
| Li/ 2006 | China | A |  |  | A |  |  |  |  |  |  |
| Liu/ 2019 | China | A | A |  |  |  |  |  |  |  |  |
| Madeleine/ 2000 | USA | C |  |  |  | C (NA) |  | C | C |  | C |
| Makni/ 2000 | Brazil | C(HWD) |  |  |  |  |  | C |  |  | C (HWD) |
| Malcolm/ 2000 | USA | C |  |  |  |  |  |  | C (NA) |  | C (NA) |
| Malisic/ 2013 | Serbia | C | C |  |  |  |  |  |  |  |  |
| Minaguchi/ 1998 | Japan | A |  |  |  | A (NA) | A (NA) | A |  |  | A |
| Min-min/ 2006 | China | A |  |  |  | A (NA) |  | A |  |  |  |
| Mitra/ 2005 | India | I |  |  |  |  | I (NA) | I | I |  |  |
| Mostaid/ 2021 | Bangladesh | A(HWD) | A |  |  |  |  |  |  |  |  |
| Nagpal/ 2002 | India | I |  |  |  | I (NA) | I (NA) | I | I |  | I (HWD) |
| Ndiaye/ 2014 | Senegal | Af |  | Af (NA) |  |  |  |  |  |  |  |
| Ngan/ 1999 | China | A |  |  | A (HWD) | A (NA) | A (NA) | A (HWD) | A (NA) |  | A (HWD) |
| Nishikawa/ 2000 | Japan | A |  |  |  | A (NA) |  |  | A (NA) |  | A (NA) |
| Niwa/ 2004 | Japan | A | A |  |  |  |  | A | A |  |  |
| Ojeda/ 2003 | Chile | C |  |  |  |  |  | C |  |  |  |
| Pegoraro/ 2000 | South Africa | Af |  |  |  |  |  | Af |  |  | Af |
| Pegoraro/ 2002 | South Africa | Af |  | Af (NA) |  | Af (NA) |  | Af | Af |  | Af |
| Pillai/ 2002 | India | I |  |  |  | A (NA) |  | I | I |  | I |
| Piña-Sánchez/ 2010 | Mexico (Mestizo) | M |  |  |  | M (NA) |  |  |  |  |  |
| Qie/ 2002 | China | A |  |  | A |  |  |  |  |  |  |
| Ratre/ 2019 | India | I | I |  |  |  |  |  |  |  |  |
| Rezza/ 2001 | Italy | C |  |  |  |  |  |  | C | C (NA) | C |
| Rosenthal/ 1998 | UK | C |  |  |  |  |  | C | C | C (NA) | C |
| Santos/ 2006 | Portugal | C |  |  |  |  |  | C |  |  |  |
| Santos/ 2005 | Portugal | C(HWD) | C |  |  |  |  | C |  | C (NA) |  |
| Saranath/ 2002 | India | I | I (NA) |  |  | I (NA) | I (NA) | I | I (HWD) |  | I (HWD) |
| Settheetham-Ishida/ 2004 | Thailand | C |  |  |  | C (NA) |  | A | A |  |  |
| Singhal/ 2013 | India | I | I |  |  |  |  |  |  |  |  |
| Sonoda/ 1999 | USA | C |  |  |  |  |  | C |  |  | C |
| Storey/ 1998 | UK | C |  |  |  | C (NA) |  | C |  | C (NA) | C (HWD) |
| Strickler/ 1998 | Jamaica | Af |  |  |  |  |  | Af |  |  | Af |
| Suárez-Rincón/ 2002 | Mexico | M |  |  |  |  |  |  | M (HWD) |  | M (HWD) |
| Szarka/ 1999 | Hungary | C(HWD) |  |  |  | C (NA) |  | C | C | C (NA) | C |
| Tachezy/ 1999 | Czech Republic | C |  |  |  | C (NA) |  | C | C | C (NA) | C |
| Tanara/ 2003 | Gambia | Af(HWD) |  | Af (NA) |  |  |  |  |  |  |  |
| Tenti/ 2000 | Italy | C |  |  |  | C (NA) |  | C | C | C (NA) | C |
| Tong/ 2000 | Austria | C |  |  |  |  |  |  |  | C (NA) |  |
| Ueda/ 2006 | Japan | A |  |  |  | A (NA) |  | A | A |  |  |
| Ueda/ 2010 | Japan | A |  |  |  | A (NA) |  |  |  |  |  |
| Van Duin/ 2000 | Netherlands | C |  |  |  | C (NA) |  | C | C | C (NA) | C |
| Wang/ 2004 | China | A |  |  | A |  | A (NA) |  |  |  | A |
| Wu/ 2004 | China | A |  |  | A |  | A (NA) | A | A |  |  |
| Yamashita/ 1999 | Japan | A(HWD) |  |  |  | A (NA) |  | A | A |  | A |
| Yang/ 2011 | China | A(HWD) |  |  | A |  | A (NA) |  |  |  |  |
| Yang/ 2001 | China | A |  |  | A | A (NA) |  |  |  |  |  |
| Yang/ 2008 | China | A |  |  |  |  | A (NA) |  |  |  |  |
| Yao/ 2008 | China | A(HWD) |  |  | A |  | A (NA) |  |  |  |  |
| Ye/ 2010 | China | A | A |  |  | A (NA) |  | A |  |  |  |
| Yuan/ 2016 | China | A | A |  |  |  |  |  |  |  |  |
| Zehbe/ 1999 | Sweden | C |  |  |  |  |  | C |  | C (NA) | C |
| Zehbe/ 2001 | Sweden | C |  |  |  | C (NA) |  | C | C | C (NA) | C |
| Zheng/ 2008 | China | A(HWD) |  |  | A | A (NA) |  |  |  |  |  |
| Zhou/ 2009 | China | A | A |  | A |  |  |  |  |  |  |
