# Supplementary material for: Association between the p53 polymorphisms and cervical cancer risk: an updated meta-analysis
Source: Front Oncol. 2025 Feb 21;15:1461737. doi: 10.3389/fonc.2025.1461737 (PMC11885137; doi:10.3389/fonc.2025.1461737)
Supplement: Supplementary file 1 [file DataSheet1.zip › Supplementary Table 3.DOCX]

| **S3 Table. Results of published meta-analyses between *P53 rs1042522* polymorphisms and cervical cancer risk** | | | | | | | | | | | | | | |
| --- | --- | --- | --- | --- | --- | --- | --- | --- | --- | --- | --- | --- | --- | --- |
| allele (C)Pro as the effect allele | | | | | | | | | | | | | | |
| First author/year | Variable | n (Cases/Controls) | Pro/Pro + Arg/Pro vs Arg/Arg | | Pro/Pro vs Arg/Arg + Arg/Pro | | Arg/Pro vs Arg/Arg | | Pro/Pro vs Arg/Arg | | Pro vs Arg | | additive models | |
|  |  |  | OR (95% CI) | *Ph/I^2^ (%)* | OR (95% CI) | *Ph/I^2^ (%)* | OR (95% CI) | *Ph/I^2^ (%)* | OR (95% CI) | *Ph/I^2^ (%)* | OR (95% CI) | *Ph/I^2^ (%)* | OR (95% CI) | *P*h/*I*^2^ (%) |
| Yu [7]2022 | Overall | 13 | 1.759 (1.192- 2.596) | NA/82.3 | 0.806 (0.626-1.037) | NA/0.0 | 1.534 (0.885-2.658) | NA/76.0 | 1.283 (0.874-1.885) | NA/68.7 | 0.927 (0.546-1.572) | NA/91.9 | NA | NA |
|  | Asian | 9 | 1.817 (1.172-2.814) | NA/84.6 | 0.806 (0.626-1.037) | NA/0.0 | 1.782 (0.923-3.439) | NA/81.8 | 1.429 (0.906-2.255) | NA/76.2 | 0.765 (0.354-1.651) | NA/94.7 | NA | NA |
|  | European | 1 | NA | NA | NA | NA | 0.518 (0.235-1.141) | NA | NA | NA | 2.222 (0.872-5.664) | NA | NA | NA |
|  | Other | 3 | 1.350 (0.678-2.690) | NA | NA | NA | 1.813 (0.532-6.180) | NA/42.5 | 0.751 (0.349-1.618) | NA/0.0 | 1.064 (0.763-1.484) | NA/0.0 | NA | NA |
|  | PB | 5 | 1.676 (0.605-4.646) | NA/85.6 | 0.840 (0.589-1.198) | NA | 1.143 (0.659-1.983) | NA/0.0 | 0.910 (0.671-1.235) | NA/0.0 | 1.075 (0.841-1.372) | NA/0.0 | NA | NA |
|  | HB | 8 | 1.795 (1.133-2.844) | NA/84.0 | 0.772 (0.538-1.106) | NA/0.0 | 1.711 (0.830-3.528) | NA/83.2 | 1.610 (0.859-3.020) | NA/77.7 | 0.724 (0.271-1.935) | NA/95.9 | NA | NA |
|  | High | 7 | 1.707 (1.050-2.773) | NA/74.3 | 0.789 (0.600-1.035) | NA/0.0 | 2.677 (1.317-5.444) | NA/73.0 | 1.122 (0.753-1.671) | NA/49.9 | 0.845 (0.440-1.621) | NA/93.8 | NA | NA |
|  | Moderate | 6 | 1.782 (0.899-3.533) | NA/87.2 | 0.920 (0.468-1.810) | NA | 0.954 (0.718-1.267) | NA/0.0 | 1.547 (0.668-3.580) | NA/75.7 | 1.238 (0.487-3.150) | NA/72.0 | NA | NA |
|  | Yes | 7 | 1.654 (0.827-3.307) | NA/90.9 | 0.789 (0.600-1.035) | NA/0.0 | 1.369 (0.749-2.503) | NA/69.6 | 1.136 (0.794-1.625) | NA/65.6 | 0.766 (0.420-1.396) | NA/86.7 | NA | NA |
|  | NR | 6 | 1.836 (1.084-3.110) | NA/77.0 | 0.920 (0.468-1.810) | NA | 1.857 (0.548-6.295) | NA/88.3 | 1.572 (0.723-3.417) | NA/73.5 | 1.042 (0.464-2.340) | NA/93.8 | NA | NA |
| Kamiza[8]2020 | Overall | 8(699/1008) | 0.57(0.44-0.75) | 0.45/0.0 | 0.83(0.68-1.04) | 0.70/0.0 | NA | NA | 0.62(0.46-0.83) | 0.62/0.0 | 1.30 (1.12, 1.50) | 0.32/14 | NA | NA |
| Li [9]2015 | Overall | 16(1684/1178) | 0.81 (0.58-1.14) | < 0.001 | 1.17 (0.87-1.58) | 0.004 | NA | NA | 1.05 (0.70-1.58) | < 0.001 | 0.95 (0.75-1.20) | < 0.001 | NA | NA |
|  | PB | 6(755/868) | 0.59 (0.31-1.10) | < 0.001 | 0.75 (0.46-1.21) | 0.036 | NA | NA | 0.62 (0.30-1.27) | 0.001 | 0.68 (0.44-1.05) | < 0.001 | NA | NA |
|  | HB | 10(929/714) | 0.97 (0.64-1.47) | 0.001 | 1.55 (1.20-1.99) | 0.215 | NA | NA | 1.43 (0.88-2.31) | 0.009 | 1.14 (0.86-1.50) | < 0.001 | NA | NA |
|  | Han | 6(856/823) | 1.18 (0.71-1.97) | 0.001 | 1.31 (1.05-1.63) | 0.054 | NA | NA | 1.57 (0.88-2.81) | 0.008 | 1.21 (0.88-1.67) | 0.001 | NA | NA |
|  | Uigur | 2(225/136) | 0.50 (0.32-0.78) | 0.943 | 0.94 (0.52-1.71) | 0.77 | NA | NA | 0.61 (0.32-1.19) | 0.842 | 0.70 (0.51-0.95) | 0.913 | NA | NA |
|  | Not stated | 7(550/558) | 0.64 (0.36-1.16) | < 0.001 | 1.04 (0.55-1.97) | 0.003 | NA | NA | 0.84 (0.39-1.81) | 0.001 | 0.80 (0.51-1.26) | < 0.001 | NA | NA |
| Habbous [10]2012 | HPV (+) | 18 | 1.09(0.75-1.59) | 0.64 | 1.06(0.77-1.47) | 0.72 | NA | NA | NA | NA | NA | NA | 0.90 (0.71–1.14) | 0.37 |
|  | HPV (-) | 18 | 0.93(0.60-1.45) | 0.76 | 0.89(0.69-1.16) | 0.41 | NA | NA | NA | NA | NA | NA | 0.90 (0.73–1.10) | 0.29 |
| Zhou [11] 2012 | Overall | 28(3580/3827) | 0.84 (0.70-1.01) | <0.001 | 1.13 (0.92-1.39) | <0.001 | NA | NA | 0.97（0.76-1.22) | <0.001 | 0.97(0.85-1.10) | <0.001 | NA | NA |
|  | China | 11 | 0.77 (0.57-1.04) | 0.002 | 1.16 (0.85-1.58) | 0.028 | NA | NA | 0.95 (0.67-1.36) | 0.021 | 0.93 (0.75-1.15) | <0.001 | NA | NA |
|  | Korea | 6 | 0.98 (0.68-1.40) | 0.026 | 0.88 (0.66-1.18) | 0.963 | NA | NA | 0.91 (0.67-1.24) | 0.874 | 0.97 (0.78-1.20) | 0.146 | NA | NA |
|  | Japan | 5 | 1.11 (0.78-1.58) | 0.044 | 1.28 (0.65-2.52) | <0.001 | NA | NA | 1.35 (0.63-2.89) | <0.001 | 1.18 (0.82-1.71) | <0.001 | NA | NA |
|  | India | 5 | 0.54 (0.36-0.81) | 0.234 | 1.22 (0.74-2.00) | 0.068 | NA | NA | 0.69 (0.35-1.38) | 0.035 | 0.86 (0.63-1.15) | 0.05 | NA | NA |
|  | English | NA | 0.9 (0.75-1.07) | 0.002 | 1.13 (0.91-1.40) | 0.001 | NA | NA | 0.99 (0.78-1.25) | 0.004 | 1 (0.88-1.13) | <0.001 | NA | NA |
|  | Chinese | NA | 0.66 (0.36-1.19) | 0.002 | 1.17 (0.62-2.22) | 0.03 | NA | NA | 0.89 (0.38-2.08) | 0.002 | 0.86 (0.52-1.42) | <0.001 | NA | NA |
|  | HWE | NA | 0.9 (0.73-1.11) | <0.001 | 1.08 (0.84-1.38) | <0.001 | NA | NA | 0.97 (0.72-1.29) | <0.001 | 0.98 (0.84-1.15) | <0.001 | NA | NA |
|  | Non-HWE | NA | 0.68 (0.47-0.97) | 0.08 | 1.31 (0.98-1.75) | 0.424 | NA | NA | 0.98 (0.66-1.45) | 0.251 | 0.93 (0.73-1.17) | 0.071 | NA | NA |
| Francisco [12]2010 | Overall | 22 | 0.85 (076–1.03) | NA/71.4 | 1.01 (0.94–1.26) | NA/17.0 | NA | NA | 0.84 (0.71–1.02) | NA/37.6 | NA | NA | NA | NA |
|  | Caucasian | 22(1592/3883) | 0.77 (0.68–0.88) | NA | 0.78 (0.60–1.01) | NA | NA | NA | 0.70 (0.54–0.91) | NA | NA | NA | NA | NA |
|  | Asian | 15(1924/3087） | 0.91 (0.65– 1.19) | NA | 0.82 (0.70–0.96) | NA | NA | NA | 0.85 (0.60–1.22) | NA | NA | NA | NA | NA |
|  | Indian | 7(786/760） | 0.62 (0.48–0.80) | NA | 1.23 (0.95–1.58) | NA | NA | NA | 0.84 (0.61–1.15) | NA | NA | NA | NA | NA |
|  | African | 4(462/643) | 0.93 (0.63–1.27) | NA | 0.91 (0.70–1.19) | NA | NA | NA | 0.84 (0.57–1.22) | NA | NA | NA | NA | NA |
|  | Mixed Population | 18(1380/1993) | 0.91 (0.78–1.05) | NA | 1.32 (1.05–1.66) | NA | NA | NA | 1.23 (0.95–.58) | NA | NA | NA | NA | NA |
| Klug [13]2009 | HPV Positive | 4(273/176) | 0.76(0.47-1.20) | NA | NA | NA | 0.73(0.44-1.20) | NA | 1.37(0.83-2.72) | NA | NA | NA | NA | NA |
|  | High-risk HPV positive | 4(264/134) | 0.69(0.41-1.18) | NA | NA | NA | 0.69(0.40-1.19) | NA | 1.45(0.84-2.50) | NA | NA | NA | NA | NA |
|  | White | 3(150/310) | 0.97(0.65-1.45) | NA | NA | NA | 0.88(0.58-1.37) | NA | 1.14(0.73-1.72) | NA | NA | NA | NA | NA |
|  | Asian | 1(111/114) | 0.69(0.36-1.33) | NA | NA | NA | 0.72(0.37-1.45) | NA | 1.39(0.69-2.70) | NA | NA | NA | NA | NA |
|  | Hispanic | 1(30/205) | 0.92(0.42-2.00) | NA | NA | NA | 0.80(0.34-1.85) | NA | 1.25(0.54-2.94) | NA | NA | NA | NA | NA |
|  | Invasive cervical cancer | 5(400/740) | 0.95(0.73-1.25) | NA | NA | NA | 0.93(0.70-1.23) | NA | 1.08(0.81-1.43) | NA | NA | NA | NA | NA |
|  | Squamous cell carcinoma | 4(331/535) | 0.97(0.72-1.30) | NA | NA | NA | 0.99(0.72-1.35) | NA | 1.01(0.74-1.39) | NA | NA | NA | NA | NA |
|  | Other histology | 2(21/336) | 0.87(0.36-2.13) | NA | NA | NA | 0.83(0.32-2.17) | NA | 1.20(0.46-1.13) | NA | NA | NA | NA | NA |
| Sousa [14]2007 | Overall | 24 | 0.79(0.68-0.90) | NA | NA | NA | NA | NA | NA | NA | NA | NA | NA | NA |
| Koushik [15] 2004 | invasive cervical cancer | 24 | 0.92(0.77-1.11) | NA | NA | NA | NA | NA | NA | NA | NA | NA | NA | NA |
|  | squamous cell | 22 | 0.68(0.53-0.83) | NA | NA | NA | NA | NA | NA | NA | NA | NA | NA | NA |
|  | cervical adenocarcinoma | 4 | 0.60(0.37-1.00) | NA | NA | NA | NA | NA | NA | NA | NA | NA | NA | NA |
| allele (G)Arg as the effect allele | | | | | | | | | | | | |  |  |
| First author/year | Variable | n (Cases/Controls) | Arg/Arg + Arg/Pro vs Pro/Pro | | Arg/Arg vs Arg/Pro + Pro/Pro | | Arg/Pro vs Arg/Arg | | Arg/Arg vs Pro/Pro | | Pro vs Arg | | additive models | |
|  |  |  | OR (95% CI) | *Ph/I^2^ (%)* | OR (95% CI) | *Ph/I^2^ (%)* | OR (95% CI) | *Ph/I^2^ (%)* | OR (95% CI) | *Ph/I^2^ (%)* | OR (95% CI) | *Ph/I^2^ (%)* | OR (95% CI) | *P*h/*I*^2^ (%) |
| Yu [7]2022 | Overall | 8 | 1.932 (0.821-4.547) | NA/86.5 | 1.049 (0.690-1.595) | NA/0.0 | 1.534 (0.885-2.658) | NA/76.0 | 2.442 (1.433-4.162) | NA/79.2 | NA | NA | NA | NA |
|  | Asian | 5 | 1.749 (0.560-5.463) | NA/92.8 | NA | NA | 1.782 (0.923-3.439) | NA/81.8 | 2.576 (1.259-5.271) | NA/86.1 | NA | NA | NA | NA |
|  | European | 1 | 4.240 (0.493-36.503) | NA | 1.240 (0.590-2.608) | NA | 0.518 (0.235-1.141) | NA | NA | NA | NA | NA | NA | NA |
|  | Other | 2 | NA | NA | 0.970 (0.584-1.611) | NA | 1.813 (0.532-6.180) | NA/42.5 | 2.051 (0.807-5.213) | NA/0.0 | NA | NA | NA | NA |
|  | PB | 2 | NA | NA | 0.970 (0.584-1.611) | NA | 1.143 (0.659-1.983) | NA/0.0 | 2.170 (1.159-4.061) | NA/0.0 | NA | NA | NA | NA |
|  | HB | 6 | 1.932 (0.821-4.547) | NA/86.5 | 1.240 (0.590-2.608) | NA | 1.711 (0.830-3.528) | NA/83.2 | 2.589 (1.239-5.410) | NA/86.1 | NA | NA | NA | NA |
|  | High | 4 | 3.213 (1.987-5.196) | NA/0.0 | 1.049 (0.690-1.595) | NA/0.0 | 2.677 (1.317-5.444) | NA/73.0 | 3.934 (2.210-7.001) | NA/53.5 | NA | NA | NA | NA |
|  | Moderate | 4 | 0.990 (0.690-1.420) | NA | NA | NA | 0.954 (0.718-1.267) | NA/0.0 | 1.231 (0.872-1.739) | NA/0.0 | NA | NA | NA | NA |
|  | Yes | 5 | 3.167 (1.934-5.185) | NA | NA | NA | 1.369 (0.749-2.503) | NA/69.6 | 2.509 (1.465-4.298) | NA/61.8 | NA | NA | NA | NA |
|  | NR | 3 | 1.369 (0.418-4.485) | NA/41.4 | 1.049 (0.690-1.595) | NA/0.0 | 1.857 (0.548-6.295) | NA/88.3 | 2.431 (0.747-7.915) | NA/89.5 | NA | NA | NA | NA |
|  |  |  |  |  |  |  |  |  |  |  |  |  |  |  |
