# Supplementary material for: Association between the p53 polymorphisms and cervical cancer risk: an updated meta-analysis
Source: Front Oncol. 2025 Feb 21;15:1461737. doi: 10.3389/fonc.2025.1461737 (PMC11885137; doi:10.3389/fonc.2025.1461737)
Supplement: Supplementary file 1 [file DataSheet1.zip › Supplementary Table 4.DOCX]

| **S4 Table General characteristic and the results of the included meta-analyses on the P53 rs1042522 polymorphism with cervical cancer risk** | | | | | | | | | | | | | | |  |
| --- | --- | --- | --- | --- | --- | --- | --- | --- | --- | --- | --- | --- | --- | --- | --- |
| **First author/Year** | **Country** | **Geographic region** | **Ethnicity** | **Type of controls** | **Matching** | **Source of genotyping material of case** | **Adjustments** | **HWE/HWD** | **Genotypes of cases** | | | **Genotypes of controls** | | |  |
|  |  |  |  |  |  |  |  |  |  |  |  |  |  |  |  |
|  |  |  |  |  |  |  |  |  | **Arg/Arg** | **Arg/Pro** | **Pro/Pro** | **Arg/Arg** | **Arg/Pro** | **Pro/Pro** |  |
| Abba [16]2003 | Argentine | South America | Mixed | Non-cancer controls | NR | Epithelial cells | NR | HWE | 7 | 9 | 2 | 115 | 134 | 28 |  |
| Agorastos [17]2000 | Greece | Europe | Caucasian | Healthy controls | Age | Exfoliated cells | NR | HWE | 8 | 3 | 1 | 32 | 33 | 11 |  |
| Alsbeih [18]2013 | Saudi Arabia | South Asia | Caucasian | Non-cancer controls | History of cancer,Age | Cervix tumor | Yes | HWE | 20 | 58 | 22 | 22 | 52 | 66 |  |
| Alsbeih [19]2017 | Saudi Arabia | South Asia | Asian | Healthy controls | Age, ethnicity, and gravida | Epithelial cells,Blood | NR | HWE | 47 | 124 | 61 | 64 | 181 | 68 |  |
| Andersson [20]2001 | Sweden | Europe | Caucasian | Healthy controls | NR | Tumour blocks | NR | HWE | 79 | 32 | 0 | 89 | 83 | 16 |  |
| Apu [21]2020 | Bangladesh | South Asia | Asian | Healthy controls | Age | Blood | Yes | HWD | 59 | 41 | 28 | 70 | 21 | 8 |  |
| Arbel-Alon [22]2002 | Israel | West Asia | Caucasian | Healthy controls | Origin | Blood | NR | HWD | 8 | 15 | 0 | 24 | 134 | 4 |  |
| Assoumou [23]2015 | Gabonese | Africa | African | Healthy controls | Age | Cervical smears | NR | HWE | 11 | 16 | 4 | 16 | 44 | 11 |  |
| Baek [24]2000 | Korea | East Asia | Asian | Healthy controls | NR | Blood | NR | HWE | 22 | 21 | 9 | 41 | 42 | 20 |  |
| Barbisan [25]2011 | Argentine | South America | Mixed | Non-cancer controls | Age | Exfoliated cells, freshly frozen tissue, paraffin-embedded tissue | Yes | HWD | 42 | 53 | 3 | 69 | 44 | 10 |  |
| Bertorelle [26]1999 | Italy | Europe | Caucasian | Healthy controls | NR | Exfoliated cells,Blood | NR | HWE | 54 | 40 | 10 | 71 | 47 | 12 |  |
| Bhattacharya [27]2002 | India | South Asia | Indian | Non-cancer controls | Age | Cervical tissues,cell scrapes | NR | HWE | 15 | 22 | 18 | 55 | 110 | 36 |  |
| Bhattacharya [28]2005 | India | South Asia | Indian | Non-cancer controls | Age | Cervical tissues,cell scrapes | Yes | HWE | 45 | 93 | 64 | 33 | 115 | 57 |  |
| Boumba [29]2017 | Congo | Africa | African | Healthy controls | NR | NR | NR | HWD | 5 | 11 | 39 | 4 | 20 | 26 |  |
| Brady [30]1999 | England | Europe | Caucasian | Non-cancer controls | Age | Peripheral Blood mononuclear cells | NR | HWE | 59 | 24 | 2 | 43 | 29 | 2 |  |
| Calhoun [31]2002 | US | North America | Caucasian | Non-cancer controls | Origin | Blood | Yes | HWE | 60 | 56 | 11 | 62 | 42 | 4 |  |
| Cenci [32]2003 | India | South Asia | Indian | Non-cancer controls | Age | Exfoliated cells | Yes | HWD | 14 | 8 | 8 | 23 | 26 | 1 |  |
| Chansaenroj [33]2013 | Thailand | South Asia | Asian | Non-cancer controls | Age | Cervical swab | NR | HWD | 17 | 16 | 23 | 24 | 28 | 16 |  |
| Chen [34]2012 | China | East Asia | Asian | Non-cancer controls | NR | Cervical tissues | NR | HWE | 22 | 83 | 51 | 22 | 34 | 16 |  |
| Cho [35]2003 | Korea | East Asia | Asian | Non-cancer controls | NR | Cervical tissues | NR | HWE | 41 | 2 | 3 | 174 | 82 | 10 |  |
| Ciotti [36]2006 | Italy | Europe | Caucasian | Non-cancer controls | NR | Exfoliated cells | NR | HWE | 47 | 9 | 2 | 19 | 10 | 3 |  |
| Comar [37]2003 | Italy | Europe | Caucasian | Non-cancer controls | Age | Exfoliated cells | Yes | HWE | 27 | 23 | 2 | 48 | 27 | 6 |  |
| Dokianakis [38]2000 | Greece | Europe | Caucasian | Healthy controls | Age,HPV | Exfoliated cell,Peripheral Blood | NR | HWE | 21 | 14 | 2 | 34 | 99 | 21 |  |
| Dybikowska [39]2000 | Poland | Europe | Caucasian | Healthy controls | NR | NR | Yes | HWE | 31 | 12 | 1 | 38 | 12 | 2 |  |
| El Khair [40]2009 | Moroccan | Africa | Caucasian | Non-cancer controls | NR | Fresh frozen biopsies of Cervical specimens | Yes | HWE | 18 | 58 | 24 | 19 | 57 | 37 |  |
| Eltahir [41]2012 | Sudan | Africa | African | Non-cancer controls | Race and Diagnosis | Blood | NR | HWE | 34 | 29 | 15 | 7 | 13 | 16 |  |
| Fernandes [42]2008 | Brazil | South America | Mixed | Healthy controls | Race | Blood,Frozen tumor | NR | HWE | 24 | 19 | 2 | 17 | 7 | 5 |  |
| Fernandes [42]2008 | Brazil | South America | Caucasian | Healthy controls | Race | Blood,Frozen tumor | NR | HWE | 13 | 13 | 2 | 11 | 4 | 3 |  |
| Ferreira da Silva [43]2010 | Brazil | South America | Mixed | Non-cancer controls | NR | Blood | Yes | HWE | 33 | 117 | 26 | 31 | 68 | 29 |  |
| Giannoudis [44]1999 | England | Europe | Caucasian | Non-cancer controls | NR | Cervical tissues | NR | HWE | 27 | 15 | 1 | 132 | 114 | 20 |  |
| González Herrera [45]2014 | Mexico | South America | Mixed | Non-cancer controls | Race | Exfoliated cells | Yes | HWE | 41 | 33 | 4 | 195 | 184 | 26 |  |
| Govan [46]2007 | South Africa | Africa | African | Non-cancer controls | Age, ethnic group and area of residence | Exfoliated cells, Blood | Yes | HWE | 3 | 16 | 13 | 3 | 12 | 7 |  |
| Govan [46]2007 | South Africa | Africa | Mixed | Non-cancer controls | Age, ethnic group and area of residence | Exfoliated cells, Blood | Yes | HWE | 16 | 38 | 25 | 20 | 61 | 40 |  |
| Gudleviciene [47]2006 | Lithuanian | Europe | Caucasian | Non-cancer controls | Age | Exfoliated cells | Yes | HWE | 38 | 79 | 24 | 16 | 70 | 11 |  |
| Guo [48]2022 | China | East Asia | Asian | Non-cancer controls | History of cancer,Age | Blood | Yes | HWE | 100 | 49 | 41 | 65 | 97 | 48 |  |
| Gustafsson [49]2001 | Sweden | Europe | Caucasian | Non-cancer controls | NR | Cervical tissues | NR | HWE | 33 | 24 | 2 | 26 | 18 | 2 |  |
| Hayes [50]1998 | Netherland | Europe | Caucasian | Healthy controls | Area | Exfoliated cells,Blood | NR | HWE | 17 | 7 | 1 | 194 | 126 | 30 |  |
| Helland [51]1998 | Norway | Europe | Caucasian | Non-cancer controls | NR | Exfoliated cells, Blood | NR | HWD | 44 | 23 | 10 | 122 | 90 | 13 |  |
| Hildesheim [52]1998 | Costa Rica | South America | Caucasian | Non-cancer controls | NR | Exfoliated cells, Blood | NR | HWE | 20 | 22 | 7 | 127 | 158 | 36 |  |
| Hou [53]2006 | China | East Asia | Asian | Non-cancer controls | NR | Cervical tissues | NR | HWE | 47 | 25 | 9 | 14 | 24 | 22 |  |
| Humbey [54]2002 | French | Europe | Caucasian | Non-cancer controls | Age | Exfoliated cells | Yes | HWD | 41 | 46 | 1 | 26 | 24 | 0 |  |
| Isakova [55]2019 | Kyrgyz | South Asia | Asian | Non-cancer controls | History of cancer,Age | Exfoliated cells | NR | HWE | 60 | 27 | 3 | 53 | 36 | 13 |  |
| Jiang [56]2001 | China | East Asia | Asian | Non-cancer controls | History of cancer and frequency,Age | Blood | Yes | HWD | 55 | 35 | 15 | 50 | 69 | 21 |  |
| Jiang [57]2010 | China | East Asia | Asian | Non-cancer controls | Age | Blood | Yes | HWE | 33 | 48 | 23 | 30 | 83 | 47 |  |
| Josefsson [58]1998 | Sweden | Europe | Caucasian | Non-cancer controls | Age | Exfoliated cells, Blood | NR | HWE | 256 | 191 | 41 | 311 | 246 | 69 |  |
| Katiyar [59]2003 | India | South Asia | Indian | Healthy controls | Family history of cancer, Age | Exfoliated cells, Blood | NR | HWE | 48 | 86 | 29 | 12 | 49 | 13 |  |
| Kawamata [60]2002 | Japan | East Asia | Asian | Non-cancer controls | NR | Exfoliated cells | NR | HWE | 21 | 18 | 5 | 56 | 79 | 21 |  |
| Kim [61]2000 | Korea | East Asia | Asian | Healthy controls | Gender,no history of cancer,Age | Blood | Yes | HWD | 67 | 98 | 16 | 86 | 74 | 21 |  |
| Kim [62]2001 | Korea | East Asia | Asian | Healthy controls | Age | exfoliated cells | NR | HWE | 52 | 37 | 11 | 110 | 100 | 24 |  |
| Klaes [63]1999 | Germany | Europe | Caucasian | Non-cancer controls | NR | Exfoliated cells | NR | HWE | 46 | 33 | 8 | 84 | 57 | 10 |  |
| Klug [64]2001 | Peru | South America | Mixed | Non-cancer controls | Age | Exfoliated cells, tumor biopsies | Yes | HWE | 60 | 45 | 14 | 66 | 45 | 16 |  |
| Kouamou [65]2016 | Zimbabwe | Africa | African | Non-cancer controls | NR | Exfoliated cells | NR | HWE | 30 | 32 | 11 | 34 | 21 | 7 |  |
| Koushik [66]2005 | Canada | North America | Caucasian | Healthy controls | Age | Exfoliated cells | Yes | HWE | 197 | 130 | 30 | 396 | 294 | 70 |  |
| Lanham [67]1998 | UK | Europe | Caucasian | Non-cancer controls | NR | NR | NR | HWE | 20 | 15 | 3 | 273 | 208 | 34 |  |
| Laprano [68]2014 | Brazil | South America | Mixed | Non-cancer controls | NR | Cervical tissues,Blood | NR | HWE | 20 | 20 | 5 | 31 | 45 | 12 |  |
| Lee [69]2004 | Korea | East Asia | Asian | Healthy controls | NR | Exfoliated cells,Blood | Yes | HWE | 78 | 83 | 26 | 126 | 161 | 58 |  |
| Lee [70]2004 | Korea | East Asia | Asian | Non-cancer controls | NR | Exfoliated cells | Yes | HWE | 41 | 29 | 11 | 45 | 32 | 9 |  |
| Li [71]2006 | China | East Asia | Asian | Healthy controls | NR | Cervical tissues | NR | HWE | 11 | 17 | 5 | 11 | 31 | 9 |  |
| Li [72]2004 | China | East Asia | Asian | Non-cancer controls | NR | Cervical tissues | NR | HWD | 26 | 10 | 10 | 60 | 17 | 7 |  |
| Liu [73]2019 | China | East Asia | Asian | Healthy controls | NR | Blood | NR | HWE | 50 | 50 | 21 | 37 | 51 | 20 |  |
| Madeleine [74]2000 | US | Europe | Caucasian | Healthy controls | Age,Race | Blood | NR | HWE | 62 | 7 | 62 | 91 | 11 | 62 |  |
| Makni [75]2000 | Brazil | South America | Mixed | Non-cancer controls | Age | Exfoliated cells | Yes | HWD | 49 | 40 | 20 | 53 | 119 | 22 |  |
| Malcolm [76]2000 | US | Europe | Caucasian | Non-cancer controls | NR | Cervical tissues | NR | HWE | 72 | 68 | 18 | 120 | 115 | 29 |  |
| Malisic [77]2013 | Serbia | Europe | Caucasian | Non-cancer controls | Age | Cervical tissues | NR | HWE | 31 | 17 | 1 | 43 | 25 | 6 |  |
| Minaguchi [78] 1998 | Japan | East Asia | Asian | Healthy controls | NR | Cervical tissues,Blood | NR | HWE | 72 | 85 | 20 | 40 | 51 | 19 |  |
| Min-min [79]2006 | China | East Asia | Asian | Non-cancer controls | NR | Cervical tissues | NR | HWE | 47 | 25 | 9 | 14 | 24 | 22 |  |
| Mitra [80]2005 | India | South Asia | Indian | Non-cancer controls | Race | Cervical tissues,Blood | NR | HWE | 19 | 27 | 12 | 14 | 51 | 29 |  |
| Mostaid [81]2021 | Bangladesh | South Asia | Asian | Healthy controls | Age | Blood | Yes | HWD | 60 | 41 | 28 | 84 | 27 | 11 |  |
| Nagpal [82]2002 | India | South Asia | Indian | Non-cancer controls | Age | Tumour tissues | NR | HWE | 21 | 37 | 13 | 14 | 41 | 13 |  |
| Natphopsuk [83]2012 | Thailand | South Asia | Asian | Healthy controls | Age | Exfoliated cells | Yes | HWE | 57 | 81 | 39 | 49 | 76 | 52 |  |
| Ndiaye [84]2014 | Senegal | Africa | African | Healthy controls | NR | Blood | NR | HWE | 4 | 10 | 16 | 12 | 33 | 42 |  |
| Ngan [85]1999 | China Hong Kong | East Asia | Asian | Healthy controls | NR | Cervical tissues | NR | HWE | 31 | 50 | 21 | 15 | 45 | 8 |  |
| Nishikawa [86]2000 | Japan | East Asia | Asian | Non-cancer controls | NR | Exfoliated cells | NR | HWE | 39 | 10 | 38 | 11 | 6 | 11 |  |
| Niwa [87]2004 | Japan | East Asia | Asian | Healthy controls | Gender,no history of cancer | Blood | Yes | HWE | 44 | 51 | 17 | 178 | 210 | 54 |  |
| Ojeda [88]2003 | Chile | South America | Mixed | Healthy controls | No history of cancer | Cervical cells,Blood | NR | HWE | 41 | 15 | 4 | 25 | 24 | 4 |  |
| Pegoraro [89]2000 | South Africa | Africa | Mixed | Healthy controls | Race | Blood | NR | HWE | 20 | 56 | 45 | 27 | 108 | 116 |  |
| Pegoraro [90]2002 | South Africa | Africa | Mixed | Healthy controls | Race | Cervical tissues | NR | HWE | 44 | 123 | 114 | 32 | 147 | 161 |  |
| Pillai [91]2002 | India | South Asia | Indian | Non-cancer controls | NR | Blood, Exfoliated cells, and Cervical biopsies | NR | HWE | 47 | 112 | 73 | 35 | 97 | 57 |  |
| Piña-Sánchez [92]2010 | Mexico | South America | Mixed | Healthy controls | Race | Exfoliated cells Blood | NR | HWE | 66 | 35 | 10 | 92 | 30 | 4 |  |
| Qie [93]2002 | China | East Asia | Asian | Non-cancer controls | NR | Tumour blocks,Blood | NR | HWE | 12 | 2 | 1 | 8 | 11 | 1 |  |
| Ratre [94]2019 | India | North America | Indian | Healthy controls | Age | Blood | Yes | HWE | 49 | 37 | 14 | 25 | 22 | 53 |  |
| Rezza [95]2001 | Italy | Europe | Caucasian | Healthy controls | NR | Exfoliated cells | Yes | HWE | 35 | 25 | 11 | 86 | 71 | 15 |  |
| Rosenthal [96]1998 | UK | Europe | Caucasian | Non-cancer controls | NR | Cervical tissues,Blood | NR | HWE | 27 | 20 | 3 | 154 | 76 | 16 |  |
| Santos [97]2005 | Portugal | Europe | Caucasian | Non-cancer controls | Age | Blood | NR | HWE | 110 | 47 | 7 | 135 | 64 | 21 |  |
| Santos [98]2006 | Portugal | Europe | Caucasian | Healthy controls | NR | Blood | Yes | HWD | 250 | 97 | 24 | 117 | 58 | 13 |  |
| Saranath [99]2002 | India | South Asia | Indian | Healthy controls | Race | Blood | NR | HWE | 29 | 72 | 33 | 18 | 93 | 20 |  |
| Settheetham-Ishida [100]2004 | Thailand | South Asia | Asian | Healthy controls | Age | Exfoliated cells | Yes | HWE | 23 | 50 | 17 | 22 | 53 | 25 |  |
| Settheetham-Ishida [101]2005 | Thailand | South Asia | Asian | Healthy controls | Age | Exfoliated cells | NR | HWE | 23 | 50 | 17 | 22 | 53 | 25 |  |
| Singhal [102]2013 | India | South Asia | Indian | Healthy controls | Age,ethnicity | Cervical tissue | Yes | HWE | 64 | 88 | 30 | 30 | 82 | 70 |  |
| Sonoda [103]1999 | US | North America | Caucasian | Non-cancer controls | NR | Tumour blocks,Blood | NR | HWE | 50 | 41 | 14 | 55 | 35 | 10 |  |
| Storey [104]1998 | uk | Europe | Caucasian | Non-cancer controls | NR | Tumour blocks,Blood | NR | HWE | 23 | 5 | 2 | 15 | 24 | 2 |  |
| Strickler [105]1998 | Jamaica | North America | African | Healthy controls | Frequency,Age | Blood | NR | HWE | 6 | 11 | 11 | 4 | 13 | 13 |  |
| Suárez-Rincón [106]2002 | Mexico | South America | Mixed | Non-cancer controls | NR | Tumour blocks,Blood | NR | HWE | 17 | 19 | 2 | 16 | 32 | 4 |  |
| Szarka [107]1999 | Hungary | Europe | Caucasian | Healthy controls | NR | Blood | NR | HWD | 52 | 22 | 8 | 52 | 31 | 4 |  |
| Tachezy [108]1999 | Czech | Europe | Caucasian | Healthy controls | NR | Cervical tissues,Blood | NR | HWE | 37 | 28 | 6 | 92 | 61 | 19 |  |
| Tanara [109]2003 | Gambia | Africa | African | Non-cancer controls | Race | Cervical tissues | NR | HWE | 8 | 4 | 28 | 4 | 4 | 12 |  |
| Tenti [110]2000 | Italy | Europe | Caucasian | Healthy controls | Age,Ethnicity | Tumour blocks,Blood | NR | HWE | 57 | 35 | 9 | 86 | 47 | 7 |  |
| Tong [111]2000 | Austrian | Europe | Caucasian | Healthy controls | Ethnicity | Blood | NR | HWE | 57 | 40 | 8 | 83 | 39 | 11 |  |
| Ueda [112]2006 | Japan | East Asia | Asian | Healthy controls | NR | Blood | NR | HWE | 28 | 46 | 9 | 34 | 54 | 7 |  |
| Ueda [113]2010 | Japan | East Asia | Asian | Healthy controls | NR | Blood | NR | HWE | 28 | 46 | 9 | 150 | 172 | 52 |  |
| Van Duin [114]2000 | Netherland | Europe | Caucasian | Healthy controls | NR | Cervical tissues | NR | HWE | 44 | 24 | 3 | 49 | 34 | 3 |  |
| Wang [115]2004 | China | East Asia | Asian | Non-cancer controls | NR | Cervical tissues | NR | HWE | 8 | 15 | 15 | 13 | 16 | 5 |  |
| Wu [116]2004 | China Taiwan | East Asia | Asian | Healthy controls | NR | Cervical tissues | NR | HWE | 27 | 57 | 15 | 67 | 92 | 34 |  |
| Yamashita [117]1999 | Japan | East Asia | Asian | Non-cancer controls | NR | Tumour blocks | NR | HWD | 50 | 79 | 7 | 80 | 136 | 36 |  |
| Yang [118]2001 | China | East Asia | Asian | Healthy controls | NR | Cervical tissues | NR | HWE | 11 | 33 | 9 | 13 | 40 | 12 |  |
| Yang [119]2008 | China | East Asia | Asian | Non-cancer controls | NR | Cervical tissues | NR | HWE | 70 | 62 | 20 | 33 | 61 | 16 |  |
| Yang [120]2011 | China Taiwan | East Asia | Asian | Healthy controls | NR | Cervical tissues | NR | HWD | 30 | 30 | 20 | 25 | 43 | 20 |  |
| Yang [121]2014 | China | East Asia | Asian | Non-cancer controls | Age | Tumour blocks,blood | NR | HWE | 48 | 46 | 20 | 62 | 82 | 56 |  |
| Yao [122]2008 | China | East Asia | Asian | Non-cancer controls | NR | Cervical tissues | NR | HWD | 35 | 18 | 8 | 8 | 21 | 16 |  |
| Ye [123]2010 | China | East Asia | Asian | Healthy controls | Age | Cervical tissues,Blood | Yes | HWE | 203 | 413 | 184 | 47 | 358 | 95 |  |
| Yi [124]2017 | China | East Asia | Asian | Healthy controls | NR | Blood | NR | HWE | 60 | 73 | 34 | 56 | 73 | 31 |  |
| Yuan [125]2016 | China | East Asia | Asian | Healthy controls | Race | Cervical tissues,Blood | Yes | HWE | 93 | 200 | 35 | 194 | 293 | 81 |  |
| Zehbe [126]1999 | Italy | Europe | Caucasian | Healthy controls | NR | Tumour blocks | NR | HWE | 22 | 5 | 1 | 21 | 18 | 1 |  |
| Zehbe [126]1999 | Sweden | Europe | Caucasian | Healthy controls | NR | Tumour blocks | NR | HWD | 22 | 7 | 1 | 311 | 246 | 69 |  |
| Zehbe [127]2001 | Italy | Europe | Caucasian | Healthy controls | NR | Tumour blocks | NR | HWE | 33 | 7 | 3 | 21 | 18 | 1 |  |
| Zehbe [127]2001 | Sweden | Europe | Caucasian | Healthy controls | NR | Tumour blocks | NR | HWE | 46 | 22 | 4 | 89 | 83 | 16 |  |
| Zheng [128]2008 | China | East Asia | Asian | Non-cancer controls | NR | Cervical tissues | NR | HWD | 94 | 117 | 61 | 65 | 129 | 38 |  |
| Zhou [129]2009 | China | East Asia | Asian | Healthy controls | Age and residential areas | Cervical tissues,Blood | Yes | HWE | 123 | 206 | 75 | 118 | 198 | 88 |  |
| HWE: Hardy-Weinberg equilibrium; HWD: Hardy-Weinberg Disequilibrium | | | | |  |  |  |  |  |  |  |  |  |  |  |
