# Supplementary material for: Association between the p53 polymorphisms and cervical cancer risk: an updated meta-analysis
Source: Front Oncol. 2025 Feb 21;15:1461737. doi: 10.3389/fonc.2025.1461737 (PMC11885137; doi:10.3389/fonc.2025.1461737)
Supplement: Supplementary file 1 [file DataSheet1.zip › Supplementary Table 5.DOCX]

| **S5 Table General characteristic and the results of the included meta-analyses on the P53 rs17878362 polymorphism with cervical cancer risk** | | | | | | | | | | | | | | |  |
| --- | --- | --- | --- | --- | --- | --- | --- | --- | --- | --- | --- | --- | --- | --- | --- |
| **First author/Year** | **Country** | **Geographic region** | **Ethnicity** | **Type of controls** | **Matching** | **Source of genotyping material of case** | **Adjustments** | **HWE** | **Genotypes of cases** | | | **Genotypes of controls** | | |  |
|  |  |  |  |  |  |  |  |  |  |  |  |  |  |  |  |
|  |  |  |  |  |  |  |  | **P** | **A1/A1** | **A1/A2** | **A2/A2** | **A1/A1** | **A1/A2** | **A2/A2** |  |
| Calhoun [31]2002 | US | North America | Caucasian | Non-cancer controls | Origin | Blood | Yes | HWE | 96 | 31 | 0 | 80 | 28 | 0 |  |
| Fernandes [42]2008 | Brazil | South America | Mixed | Healthy controls | Race | Blood, Frozen tumor | NR | HWE | 44 | 8 | 0 | 26 | 5 | 1 |  |
| Fernandes [42]2008 | Brazil | South America | Caucasian | Healthy controls | Race | Blood, Frozen tumor | NR | HWE | 21 | 9 | 1 | 18 | 7 | 0 |  |
| Kim [61]2000 | Korea | Asia | Asian | Healthy controls | Gender, no history of cancer, Age | Blood | Yes | HWE | 167 | 14 | 0 | 171 | 10 | 0 |  |
| Laprano [68]2014 | Brazil | South America | Mixed | Non-cancer controls | NR | Cervical tissues, Blood | NR | HWE | 26 | 17 | 2 | 62 | 24 | 2 |  |
| Mitra [80]2005 | India | Asia | Indian | Non-cancer controls | Race | Cervical tissues, Blood | NR | HWE | 45 | 15 | 1 | 61 | 29 | 3 |  |
| Yi [124]2017 | China | Asia | Asian | Healthy controls | NR | Blood | NR | HWE | 153 | 14 | 0 | 146 | 14 | 0 |  |
| HWE: Hardy-Weinberg equilibrium; HWD: Hardy-Weinberg Disequilibrium | | | | | |  |  |  |  |  |  |  |  |  |  |
