# Supplementary material for: Association between the p53 polymorphisms and cervical cancer risk: an updated meta-analysis
Source: Front Oncol. 2025 Feb 21;15:1461737. doi: 10.3389/fonc.2025.1461737 (PMC11885137; doi:10.3389/fonc.2025.1461737)
Supplement: Supplementary file 1 [file DataSheet1.zip › Supplementary Table 6.DOCX]

| **S6 Table Scale for quality assessment of molecular association studies of cervical cancer** | | | | | | | | | | | |
| --- | --- | --- | --- | --- | --- | --- | --- | --- | --- | --- | --- |
| First author/Year | Source of case | Source of control | Ascertainment of cancer | Ascertainment of control | Matching | Source of genotyping material of case | Genotyping examination | HWE | Association assessment | Total sample size | Total score |
| Abba [16]2003 | 1 | 1 | 2 | 2 | 0 | 1 | 1 | 2 | 0 | 1 | 11 |
| Agorastos [17]2000 | 2 | 2 | 2 | 2 | 2 | 2 | 1 | 2 | 1 | 1 | 17 |
| Alsbeih [18]2013 | 1 | 2 | 2 | 2 | 2 | 2 | 1 | 2 | 1 | 2 | 17 |
| Alsbeih [19]2017 | 1 | 1 | 2 | 2 | 2 | 2 | 2 | 2 | 1 | 2 | 17 |
| Andersson [20]2001 | 2 | 2 | 2 | 2 | 0 | 2 | 2 | 2 | 1 | 2 | 17 |
| Apu [21]2020 | 3 | 2 | 2 | 2 | 2 | 2 | 1 | 0 | 2 | 2 | 18 |
| Arbel-Alon [22]2002 | 2 | 3 | 2 | 2 | 2 | 2 | 1 | 0 | 0 | 1 | 15 |
| Assoumou [23]2015 | 3 | 1 | 2 | 2 | 2 | 1 | 1 | 2 | 1 | 1 | 16 |
| Baek [24]2000 | 2 | 2 | 2 | 2 | 0 | 2 | 2 | 0 | 0 | 1 | 13 |
| Barbisan [25]2011 | 3 | 1 | 2 | 2 | 2 | 2 | 2 | 2 | 2 | 2 | 20 |
| Bertorelle [26]1999 | 2 | 2 | 2 | 2 | 0 | 2 | 2 | 2 | 0 | 2 | 16 |
| Bhattacharya [27]2002 | 3 | 1 | 2 | 1 | 2 | 2 | 1 | 2 | 2 | 1 | 17 |
| Bhattacharya [28]2005 | 3 | 2 | 2 | 2 | 2 | 2 | 2 | 2 | 0 | 2 | 19 |
| Boumba [29]2017 | 2 | 2 | 2 | 2 | 0 | 2 | 2 | 0 | 0 | 1 | 13 |
| Brady [30]1999 | 2 | 1 | 2 | 2 | 2 | 1 | 2 | 2 | 1 | 1 | 16 |
| Calhoun [31]2002 | 2 | 2 | 2 | 2 | 2 | 2 | 1 | 2 | **2** | 2 | 19 |
| Cenci [32]2003 | 2 | 2 | 2 | 2 | 2 | 1 | 1 | 0 | 1 | 1 | 14 |
| Chansaenroj [33]2013 | 3 | 1 | 2 | 2 | 2 | 2 | **2** | 0 | 1 | 1 | 16 |
| Chen [34]2012 | 2 | 3 | 2 | 2 | 0 | 2 | 2 | 2 | 0 | 2 | 17 |
| Cho [35]2003 | 2 | 2 | 2 | 2 | 0 | 1 | 2 | 2 | 1 | 2 | 16 |
| Ciotti [36]2006 | 2 | 2 | 2 | 2 | 2 | 2 | 2 | 2 | 1 | 1 | 18 |
| Comar [37]2003 | 3 | 3 | 2 | 2 | 2 | 2 | 2 | 2 | 2 | 1 | 21 |
| Dokianakis [38]2000 | 2 | 2 | 2 | 2 | 2 | 2 | 2 | 2 | 0 | 1 | 17 |
| Dybikowska [39]2000 | 2 | 1 | 2 | 2 | 0 | 2 | 2 | 2 | 1 | 1 | 15 |
| El Khair [40]2009 | 3 | 1 | 2 | 2 | 0 | 2 | 2 | 2 | 2 | 2 | 18 |
| Eltahir [41]2012 | 2 | 3 | 2 | 2 | 2 | 2 | 2 | 2 | 0 | 1 | 18 |
| Fernandes [42]2008 | 2 | 3 | 2 | 2 | 2 | 2 | 2 | 2 | 1 | 1 | 19 |
| Ferreira da Silva [43]2010 | 3 | 1 | 2 | 2 | 2 | 2 | 2 | 2 | 2 | 2 | 20 |
| Giannoudis [44]1999 | 2 | 1 | 2 | 2 | 0 | 2 | 2 | 2 | 1 | 2 | 16 |
| González Herrera [45]2014 | 3 | 3 | 2 | 2 | 2 | 2 | 1 | 2 | 2 | 2 | 21 |
| Govan [46]2007 | 2 | 1 | 2 | 2 | 2 | 2 | 1 | 2 | 2 | 1 | 17 |
| Gudleviciene [47]2006 | 3 | 3 | 2 | 2 | 2 | 2 | 1 | 2 | 2 | 2 | 21 |
| Guo [48]2022 | 2 | 1 | 2 | 2 | 2 | 2 | 1 | 2 | 2 | 2 | 18 |
| Gustafsson [49]2001 | 2 | 1 | 2 | 2 | 0 | 2 | 1 | 2 | 1 | 1 | 14 |
| Hayes [50]1998 | 2 | 1 | 2 | 2 | 2 | 2 | 2 | 2 | 0 | 2 | 17 |
| Helland [51]1998 | 3 | 1 | 2 | 2 | 0 | 2 | 2 | 0 | 1 | 2 | 15 |
| Hildesheim [52]1998 | 3 | 1 | 2 | 2 | 0 | 2 | 2 | 2 | 0 | 2 | 16 |
| Hou [53]2006 | 2 | 2 | 2 | 2 | 0 | 2 | 2 | 2 | 1 | 1 | 16 |
| Humbey [54]2002 | 3 | 3 | 2 | 2 | 2 | 2 | 2 | 0 | 2 | 1 | 19 |
| Isakova [55]2019 | 3 | 3 | 2 | 2 | 2 | 2 | 1 | 2 | 1 | 1 | 19 |
| Jiang [56]2001 | 2 | 1 | 2 | 2 | 2 | 2 | 2 | 0 | 2 | 2 | 17 |
| Jiang [57]2010 | 2 | 1 | 2 | 2 | 2 | 2 | 1 | 2 | 2 | 2 | 18 |
| Josefsson [58]1998 | 3 | 1 | 2 | 2 | 2 | 2 | 2 | 2 | 0 | 3 | 19 |
| Katiyar [59]2003 | 3 | 1 | 2 | 2 | 2 | 2 | 2 | 2 | 0 | 2 | 18 |
| Kawamata [60]2002 | 2 | 2 | 2 | 2 | 0 | 2 | 2 | 2 | 0 | 2 | 16 |
| Kim [61]2000 | 2 | 3 | 2 | 1 | 2 | 2 | 2 | 0 | 2 | 2 | 18 |
| Kim [62]2001 | 2 | 1 | 2 | 2 | 2 | 2 | 2 | 2 | 0 | 2 | 17 |
| Klaes [63]1999 | 2 | 1 | 2 | 2 | 0 | 2 | 2 | 2 | 0 | 2 | 15 |
| Klug [64]2001 | 2 | 1 | 2 | 2 | 2 | 2 | 2 | 2 | 2 | 2 | 19 |
| Kouamou [65]2016 | 2 | 2 | 2 | 2 | 0 | 2 | 2 | 2 | 2 | 1 | 17 |
| Koushik [66]2005 | 2 | 1 | 2 | 2 | 2 | 2 | 2 | 2 | 2 | 3 | 20 |
| Lanham [67]1998 | 2 | 2 | 2 | 2 | 0 | 0 | 2 | 2 | 0 | 3 | 15 |
| Laprano [68]2014 | 2 | 2 | 2 | 2 | 0 | 2 | 2 | 2 | 0 | 1 | 15 |
| Lee [69]2004 | 2 | 1 | 2 | 2 | 0 | 2 | 2 | 2 | 2 | 3 | 18 |
| Lee [70]2004 | 2 | 1 | 2 | 2 | 0 | 2 | 2 | 2 | 2 | 1 | 16 |
| Li [71]2006 | 3 | 3 | 2 | 2 | 0 | 2 | 2 | 2 | 0 | 1 | 17 |
| Li [72]2004 | 3 | 1 | 2 | 2 | 0 | 2 | 2 | 0 | 1 | 1 | 14 |
| Liu [73]2019 | 2 | 2 | 2 | 2 | 0 | 2 | 2 | 2 | 0 | 2 | 16 |
| Madeleine [74]2000 | 3 | 3 | 2 | 1 | 2 | 2 | 2 | 2 | 0 | 2 | 19 |
| Makni [75]2000 | 2 | 1 | 2 | 2 | 2 | 2 | 2 | 0 | 1 | 2 | 16 |
| Malcolm [76]2000 | 3 | 1 | 2 | 2 | 0 | 2 | 2 | 2 | 1 | 2 | 17 |
| Malisic [77]2013 | 3 | 1 | 2 | 2 | 2 | 2 | 2 | 2 | 1 | 1 | 18 |
| Minaguchi [78] 1998 | 2 | 3 | 2 | 1 | 0 | 2 | 2 | 2 | 0 | 2 | 16 |
| Min-min [79]2006 | 2 | 1 | 2 | 2 | 0 | 2 | 2 | 2 | 0 | 1 | 14 |
| Mitra [80]2005 | 3 | 3 | 2 | 2 | 2 | 2 | 1 | 2 | 1 | 1 | 19 |
| Mostaid [81]2021 | 2 | 2 | 2 | 2 | 2 | 2 | 1 | 0 | 2 | 2 | 17 |
| Nagpal [82]2002 | 3 | 1 | 2 | 2 | 2 | 2 | 1 | 2 | 0 | 1 | 16 |
| Natphopsuk [83]2012 | 2 | 3 | 2 | 2 | 2 | 2 | 1 | 2 | 2 | 2 | 20 |
| Ndiaye [84]2014 | 2 | 1 | 2 | 1 | 0 | 2 | 1 | 2 | 1 | 1 | 13 |
| Ngan [85]1999 | 2 | 1 | 2 | 1 | 0 | 2 | 2 | 2 | 0 | 1 | 13 |
| Nishikawa [86]2000 | 2 | 1 | 2 | 2 | 0 | 2 | 1 | 2 | 0 | 1 | 13 |
| Niwa [87]2004 | 2 | 3 | 2 | 1 | 2 | 2 | 1 | 2 | 2 | 3 | 20 |
| Ojeda [88]2003 | 2 | 2 | 2 | 1 | 2 | 2 | 1 | 2 | 0 | 1 | 15 |
| Pegoraro [89]2000 | 2 | 2 | 2 | 1 | 2 | 2 | 2 | 2 | 0 | 2 | 17 |
| Pegoraro [90]2002 | 2 | 3 | 2 | 1 | 2 | 2 | 1 | 2 | 1 | 3 | 19 |
| Pillai [91]2002 | 3 | 1 | 2 | 2 | 0 | 2 | 2 | 2 | 1 | 2 | 17 |
| Piña-Sánchez [92]2010 | 3 | 1 | 2 | 2 | 2 | 2 | 2 | 2 | 0 | 2 | 18 |
| Qie [93]2002 | 2 | 1 | 2 | 2 | 0 | 2 | 2 | 2 | 0 | 1 | 14 |
| Ratre [94]2019 | 2 | 2 | 2 | 2 | 2 | 2 | 2 | 2 | 2 | 1 | 19 |
| Rezza [95]2001 | 2 | 3 | 2 | 2 | 0 | 2 | 2 | 2 | 2 | 2 | 19 |
| Rosenthal [96]1998 | 2 | 2 | 2 | 2 | 0 | 2 | 2 | 2 | 0 | 3 | 17 |
| Santos [97]2005 | 2 | 3 | 2 | 1 | 2 | 2 | 2 | 2 | 1 | 2 | 19 |
| Santos [98]2006 | 2 | 3 | 2 | 2 | 0 | 2 | 2 | 0 | 2 | 3 | 18 |
| Saranath [99]2002 | 2 | 1 | 2 | 2 | 2 | 2 | 2 | 2 | 0 | 2 | 17 |
| Settheetham-Ishida [100]2004 | 2 | 1 | 2 | 2 | 2 | 2 | 1 | 2 | 2 | 1 | 17 |
| Settheetham-Ishida [101]2005 | 3 | 3 | 2 | 2 | 2 | 2 | 1 | 2 | 0 | 1 | 18 |
| Singhal [102]2013 | 2 | 1 | 2 | 2 | 2 | 2 | 1 | 2 | 1 | 2 | 17 |
| Sonoda [103]1999 | 2 | 1 | 2 | 2 | 0 | 2 | 2 | 2 | 1 | 2 | 16 |
| Storey [104]1998 | 2 | 1 | 2 | 1 | 0 | 2 | 1 | 2 | 0 | 1 | 12 |
| Strickler [105]1998 | 2 | 2 | 2 | 2 | 2 | 2 | 2 | 2 | 2 | 1 | 19 |
| Suárez-Rincón [106]2002 | 2 | 1 | 2 | 1 | 0 | 2 | 1 | 2 | 0 | 1 | 12 |
| Szarka [107]1999 | 2 | 2 | 2 | 1 | 0 | 2 | 1 | 0 | 0 | 1 | 11 |
| Tachezy [108]1999 | 2 | 3 | 2 | 2 | 0 | 2 | 1 | 2 | 0 | 2 | 16 |
| Tanara [109]2003 | 2 | 1 | 2 | 2 | 2 | 2 | 2 | 2 | 1 | 1 | 17 |
| Tenti [110]2000 | 2 | 1 | 2 | 2 | 2 | 2 | 2 | 2 | 1 | 2 | 18 |
| Tong [111]2000 | 2 | 3 | 2 | 1 | 2 | 2 | 2 | 2 | 1 | 2 | 19 |
| Ueda [112]2006 | 2 | 3 | 2 | 2 | 0 | 2 | 2 | 2 | 1 | 1 | 17 |
| Ueda [113]2010 | 2 | 2 | 2 | 2 | 0 | 2 | 2 | 2 | 1 | 2 | 17 |
| Van Duin [114]2000 | 3 | 2 | 2 | 2 | 0 | 2 | 2 | 2 | 0 | 1 | 16 |
| Wang [115]2004 | 2 | 1 | 2 | 2 | 0 | 2 | 2 | 2 | 0 | 1 | 14 |
| Wu [116]2004 | 0 | 0 | 2 | 2 | 0 | 2 | 2 | 2 | 0 | 2 | 12 |
| Yamashita [117]1999 | 0 | 0 | 2 | 2 | 0 | 2 | 2 | 2 | 0 | 2 | 12 |
| Yang [118]2001 | 2 | 1 | 2 | 2 | 0 | 2 | 2 | 0 | 0 | 1 | 12 |
| Yang [119]2008 | 2 | 3 | 2 | 2 | 0 | 2 | 2 | 2 | 0 | 1 | 16 |
| Yang [120]2011 | 2 | 1 | 2 | 2 | 2 | 2 | 1 | 2 | 1 | 2 | 17 |
| Yang [121]2014 | 2 | 2 | 2 | 2 | 0 | 2 | 2 | 2 | 0 | 2 | 16 |
| Yao [122]2008 | 2 | 1 | 2 | 2 | 0 | 2 | 2 | 0 | 0 | 1 | 12 |
| Ye [123]2010 | 2 | 3 | 2 | 1 | 2 | 2 | 2 | 2 | 2 | 3 | 21 |
| Yi [124]2017 | 2 | 2 | 2 | 2 | 0 | 2 | 2 | 2 | 0 | 2 | 16 |
| Yuan [125]2016 | 2 | 3 | 2 | 1 | 2 | 2 | 2 | 2 | 2 | 3 | 21 |
| Zehbe [126]1999 | 2 | 1 | 2 | 2 | 0 | 2 | 2 | 2 | 0 | 3 | 16 |
| Zehbe [127]2001 | 2 | 1 | 2 | 2 | 0 | 2 | 2 | 2 | 1 | 2 | 16 |
| Zheng [128]2008 | 2 | 1 | 2 | 2 | 0 | 2 | 2 | 0 | 0 | 3 | 14 |
| Zhou [129]2009 | 3 | 2 | 2 | 2 | 2 | 2 | 2 | 2 | 2 | 3 | 22 |

HWE: Hardy-Weinberg equilibrium
